# Repurposing FDA-approved disulfiram for targeted inhibition of diphtheria toxin and the binary protein toxins of *Clostridium botulinum* and *Bacillus anthracis*

**DOI:** 10.3389/fphar.2024.1455696

**Published:** 2024-09-13

**Authors:** Joscha Borho, Merle Kögel, Amelie Eckert, Holger Barth

**Affiliations:** Institute of Experimental and Clinical Pharmacology, Toxicology and Pharmacology of Natural Products, Ulm University Medical Center, Ulm, Germany

**Keywords:** anthrax toxin, *Bacillus anthracis*, binary C2 toxin, *Clostridium botulinum*, diphtheria toxin, disulfiram, drug repositioning, transmembrane channel

## Abstract

Many bacteria act pathogenic by the release of AB-type protein toxins that efficiently enter human or animal cells and act as enzymes in their cytosol. This leads to disturbed cell functions and the clinical symptoms characteristic for the individual toxin. Therefore, molecules that directly target and neutralize these toxins provide promising novel therapeutic options. Here, we found that the FDA-approved drug disulfiram (DSF), used for decades to treat alcohol abuse, protects cells from intoxication with diphtheria toxin (DT) from *Corynebacterium diphtheria*, the causative agent of diphtheria, lethal toxin (LT) from *Bacillus anthracis*, which contributes to anthrax, and C2 enterotoxin from *Clostridium botulinum* when applied in concentrations lower than those found in plasma of patients receiving standard DSF treatment for alcoholism (up to 20 µM). Moreover, this inhibitory effect is increased by copper, a known enhancer of DSF activity. LT and C2 are binary toxins, consisting of two non-linked proteins, an enzyme (A) and a separate binding/transport (B) subunit. To act cytotoxic, their proteolytically activated B subunits PA_63_ and C2IIa, respectively, form barrel-shaped heptamers that bind to their cellular receptors and form complexes with their respective A subunits LF and C2I. The toxin complexes are internalized via receptor-mediated endocytosis and in acidified endosomes, PA_63_ and C2IIa form pores in endosomal membranes, which facilitate translocation of LF and C2I into the cytosol, where they act cytotoxic. In DT, A and B subunits are located within one protein, but DT also forms pores in endosomes that facilitate translocation of the A subunit. If cell binding, membrane translocation, or substrate modification is inhibited, cells are protected from intoxication. Our results implicate that DSF neither affects cellular binding nor the catalytic activity of the investigated toxins to a relevant extend, but interferes with the toxin pore-mediated translocation of the A subunits of DT, LT and C2 toxin, as demonstrated by membrane-translocation assays and toxin pore conductivity experiments in the presence or absence of DSF. Since toxin translocation across intracellular membranes represents a central step during cellular uptake of many bacterial toxins, DSF might neutralize a broad spectrum of medically relevant toxins.

## 1 Introduction

Many pathogenic bacteria produce protein exotoxins as their primary virulence factors, which intoxicate human and animal cells and are causative for severe and life-threatening diseases (Schmitt et al., 1999). The medically most relevant class of these toxins consist of functionally different domains. A binding (B) subunit mediates binding of the toxin to cellular receptors and receptor-mediated endocytosis. A translocation (T) subunit, in some toxins located within the B subunit, then facilitates the transport of the enzymatically active (A) subunit across intracellular membranes into the cytosol of target cells (Montecucco, 1998; Lord et al., 1999; Papatheodorou and Aktories, 2016). There, the A-subunit catalyzes the modification of specific substrate molecules, which disturbs cell morphology (Strauss and Hendee, 1959; Pappenheimer, 1977) or cell functions (Falnes and Sandvig, 2000) and is the reason for the characteristic clinical symptoms associated with the particular toxin (Pappenheimer, 1977; Schmitt et al., 1999; Moayeri et al., 2015). The toxin subunits can be either localized on one protein, as described for diphtheria toxin (DT), the main virulence factor of *Corynebacterium diphtheriae*, or on two separate proteins, as found for the binary toxins of *Clostridium* (*C*.) *botulinum* (C2 toxin) and *Bacillu*s (*B*.) *anthracis* (anthrax toxins). Here, the two individual proteins are secreted by the bacteria and must assemble in solution or on the surface of their target cells to form biologically active toxin complexes which are then internalized into cells (Popoff, 2024).

C2 toxin consists of the A-subunit C2I (∼50 kDa) (Aktories et al., 1986; Barth et al., 1998a; 1998b) and the B/T-subunit C2II (∼80 or ∼100 kDa, depending on the strain) (Ohishi, 1987; Barth et al., 2000; Sterthoff et al., 2010). C2I and C2II form biologically active C2 toxin complexes in solution or on the surface of target cells, which act cytotoxic (Ohishi, 1983a; Ohishi et al., 1984; Ohishi and Miyake, 1985; Kurazono et al., 1987; Ohishi and Yanagimoto, 1992; Barth et al., 1998a; 1998b; 2000; Stiles et al., 2014). C2II contains four functionally different domains (Schleberger et al., 2006). The N-terminal domain 1 has a cleavage site for proteolytic activation (Ohishi, 1987; Barth et al., 2000), domain 2 mediates membrane insertion and pore-formation (Blöcker et al., 2003a; Knapp et al., 2016), domain 3 binds C2I (Kaiser et al., 2006; Schleberger et al., 2006; Lang et al., 2008) and the C-terminal domain 4 mediates binding to the cell receptor (Blöcker et al., 2000). C2II is activated by limited proteolysis, where host proteases remove a ∼20 kDa peptide from the N-terminal domain of C2II. The resulting biologically active C2IIa (Ohishi, 1987; Barth et al., 2000) forms barrel-shaped heptamers (Barth et al., 2000), which bind to cells via complex and hybrid carbohydrate receptors (Eckhardt et al., 2000). C2I binds to C2IIa (Barth et al., 2000; Kaiser et al., 2006) and C2I/C2IIa complexes are taken up by cells via receptor-mediated endocytosis (Barth et al., 2000; Nagahama et al., 2009; Pust et al., 2010; Takehara et al., 2017). Subsequently, C2IIa forms pores in the membranes of acidified endosomes, which serve as translocation channels for the transport of C2I from the endosomal lumen into the cytosol (Barth et al., 2000; Bachmeyer et al., 2001; Blöcker et al., 2003b; 2003a). There, C2I catalyzes the covalent transfer of ADP-ribose from NAD (nicotinamide adenine dinucleotide) onto arginine-177 of G-actin (Aktories et al., 1986; Vandekerckhove et al., 1988), which prevents further growing of actin filaments (Aktories et al., 1989; Uematsu et al., 2007) and results in depolymerization of F-actin, cell-rounding, and cell death (Heine et al., 2008). *In vivo*, this results in a breakdown of biological barriers (Ohishi, 1983a; 1983b; Kurazono et al., 1987).

Regarding the four domain structure and mode of action, C2II is widely comparable to PA_83_, the B-subunit of the binary anthrax toxin (Barth et al., 2004; Schleberger et al., 2006; Neumeyer et al., 2008). PA_83_ is produced and secreted by *B. anthracis* and delivers two different A-subunits into the cytosol of target cells that are also produced by this bacterium: Lethal factor (LF), which cleaves MAP kinase kinases and edema factor (EF), a Ca^2+^/calmodulin-dependent adenylyl cyclase. PA plus LF forms lethal toxin, PA plus EF forms edema toxin (Young and Collier, 2007). PA_83_ has a molecular weight of 83 kDa and must be proteolytically activated to yield PA_63_ (63 kDa), the biologically active PA species (Singh et al., 1989). Like C2IIa, PA_63_ assembles into heptameric, barrel-shaped oligomers (Petosa et al., 1997; Lacy et al., 2004; Jiang et al., 2015) that form pores in endosomal membranes at low pH, which serve for the translocation of LF and EF into the cytosol (Blaustein et al., 1989; Milne and Collier, 1993; Krantz et al., 2005; Jiang et al., 2015; Hardenbrook et al., 2020). However, the C-terminally located domain 4, which binds to cells differs between C2II and PA (Schleberger et al., 2006) and PA binds to other cell surface receptors than C2IIa, namely, to anthrax toxin receptor/tumor endothelial marker 8 (Bradley et al., 2001) and anthrax toxin receptor 2/capillary morphogenesis protein 2 (Scobie et al., 2003).

Although DT (∼58 kDa) is a single chain protein toxin, its general mode of action and cellular uptake is comparable to that of the binary C2 and anthrax toxins. In particular, DT can also be subdivided into two major functional subunits. The N-terminal domain (DTA, ∼21 kDa) bears catalytic activity, while the C-terminal domain (DTB, ∼37 kDa) mediates receptor-binding and the subsequent translocation of DTA from endosomes into the cytosol of target cells (Choe et al., 1992; Papini et al., 1993; Holmes, 2000; Collier, 2001). Binding of DT to its cell surface receptor heparin-binding epidermal growth factor-like growth factor (Naglich et al., 1992; Mitamura et al., 1995) is enhanced in the presence of the co-receptor human CD9 antigen (Iwamoto et al., 1994) and initiates DT internalization into cells by receptor-mediated endocytosis (Keen et al., 1982). In the course of the uptake of DT, cellular proteases cleave the polypeptide bond between DTA and DTB (Tsuneoka et al., 1993; Chenal et al., 2002), but the subunits remain interconnected via a disulfide bond (Holmes, 2000). The low pH in endosomes then triggers a conformational change in DTB, leading to its partial insertion into the endosomal membrane and the formation of a pore (Donovan et al., 1981; Kagan et al., 1981; Blewitt et al., 1985; Papini et al., 1988; Sandvig and Olsnes, 1988) that facilitates translocation of DTA into the cytosol (Choe et al., 1992; Papini et al., 1993; Lemichez et al., 1997; Oh et al., 1999). Following translocation, the disulfide bond between DTA and DTB is reduced (Falnes and Sandvig, 2000; Murphy, 2011) and DTA is released into the cytosol, where it ADP-ribosylates the eukaryotic elongation factor 2 (Collier, 1967; Honjo et al., 1968; Collier and Cole, 1969; Van Ness et al., 1980b; 1980a) (eEF2, ∼95 kDa (Bargis-Surgey et al., 1999)). This results in an inhibition of protein biosynthesis (Collier, 1967; Honjo et al., 1968; Chenal et al., 2002), cell rounding (Strauss and Hendee, 1959) and finally cell death (Greenfield et al., 1983; Chenal et al., 2002).

Considering the increasing threat of antibiotic resistant bacteria in global health (Laxminarayan et al., 2013; Gajdács and Albericio, 2019; US Centers for Disease Control and Prevention, 2019; Uddin et al., 2021; Wickramage et al., 2021), it is of particular interest to develop novel treatment options against bacterial pathogens. One strategy is to discover therapeutic molecules directly targeting bacterial exotoxins as the symptom-causing factors in many diseases caused by pathogenic bacteria (Schmitt et al., 1999). Typically, there are three main steps in the intoxication of cells by bacterial protein toxins with intracellular targets (Papini et al., 1988) that can be targeted by therapeutic compounds: 1) cell binding, 2) intracellular membrane translocation and 3) the modulation of intracellular target molecules. Inhibiting one or multiple of these steps can prevent cells from intoxication (Bronnhuber et al., 2014; Stroke et al., 2018; Kling et al., 2023). Here, we found that the FDA-approved drug Disulfiram (DSF), which has been used for more than 60 years for the treatment of alcoholism (Wright and Moore, 1990; Suh et al., 2006; Burnette et al., 2022), protects cells from DT, C2 toxin and anthrax lethal toxin (LT). The mode of action leading to the approval of DSF for the treatment of alcohol addiction is its inhibition of aldehyde dehydrogenase. DSF administration in combination with alcohol consumption results in an accumulation of toxic acetaldehyde in the blood, accompanied by unpleasant symptoms such as nausea, vomiting and flushing (Vallari and Pietruszko, 1982; Burnette et al., 2022). However, DSF shows further activities as well. It can act as cysteine-modifying agent affecting several proteins with sulfhydryl groups (Vallari and Pietruszko, 1982; Nobel et al., 1997; Castillo-Villanueva et al., 2017; Hu et al., 2020), and is investigated for the treatment of cancer (Skrott et al., 2017; Lu et al., 2021), inflammation (Hu et al., 2020), infections, other addictions (e.g., cocaine) and neurological diseases (Lanz et al., 2023). Moreover, it is described that the presence of copper enhances the activity of DSF (Skrott et al., 2017; Hu et al., 2020). Most likely, this is due to the fact that DSF is rapidly metabolized to the active metabolite diethyldithiocarbamate (DTC), which is stabilized by complexation of copper (Skrott et al., 2017; Hu et al., 2020; Kang et al., 2023). The results presented here, however, implicate that DSF inhibits the investigated toxins by interfering with their membrane translocation, a crucial step in the intoxication process of cells that is common to a broad spectrum of bacterial protein toxins. Finally, we could also demonstrate that Cu(II) enhances the inhibitory effect of DSF towards C2 toxin and LT.

## 2 Materials and methods

### 2.1 Materials

Disulfiram (DSF; Merck, Darmstadt, Germany) was dissolved in Ethanol (EtOH; Carl Roth, Karlsruhe, Germany) for the use in all experiments. Copper (II) D-Gluconate (Cu (II); Merck, Darmstadt, Germany) was dissolved in H_2_O. Bafilomycin A1 (Santa Cruz Biotechnology, Dallas, TX, United States) was dissolved in DMSO.

### 2.2 Cell culture

HeLa cells (DSMZ, Braunschweig, Germany) and J774A.1 cells (DSMZ, Braunschweig, Germany) were cultured at constant humidity, 37°C, 5% CO_2_ and sub-cultivated every 3–4 days in a split ratio of 1:3 to 1:10 after trypsinization (HeLa cells; PAN-BIOTECH, Aidenbach, Germany) or mechanical scratching (J774A.1 cells). HeLa cells were cultivated in Minimal Essential Medium (MEM; Gibco-Life Technologies, Carlsbad, CA, United States) supplemented with 10% fetal calf serum (Gibco-Life Technologies, Carlsbad, CA, United States), 0.1 mM MEM-NEAA (Gibco-Life Technologies, Carlsbad, CA, United States), 2 mM L-Glutamin (PAN-BIOTECH, Aidenbach, GER), 1 mM sodium pyruvate (Gibco-Life Technologies, Carlsbad, CA, United States), and 1% (100 U/mL) penicillin–streptomycin (Gibco-Life Technologies, Carlsbad, CA, United States). J774A.1 cells were cultivated in Dulbecco’s Minimal Essential medium (DMEM; Gibco-Life Technologies, Carlsbad, CA, United States) supplemented with 10% fetal calf serum (Gibco-Life Technologies, Carlsbad, CA, United States), 0.1 mM MEM-NEAA (Gibco-Life Technol-ogies, Carlsbad, CA, United States), 1 mM sodium pyruvate (Gibco-Life Technologies, Carlsbad, CA, United States), and 1% (100 U/mL) penicillin–streptomycin (Gibco-Life Technologies, Carlsbad, CA, United States).

### 2.3 Cytotoxicity and cell viability assay

For cell morphology and viability assays, cells were seeded (HeLa: 6.9 × 10^4^ cells/mL, J774A.1: 11.5 × 10^4^ cells/mL) in 96 well plates (100 µL/well; Corning Incorporated, Corning, NY, United States) and grown for 1 day. Cells were treated by exchanging the cultivation medium with fresh medium (100 µL/well) containing the components indicated in the individual experiments and incubated (37°C, 5% CO_2_) for indicated time periods. In all cytotoxicity and cell viability assays, cells treated with DSF and/or Cu(II) were pre-incubated with these compounds for 30 min at 37°C before addition of the toxins. To analyze cell morphology, pictures of the cells were acquired with a LEICA MC170 HD camera (Leica Microsystems Ltd., Heerbrugg, Switzerland) connected to a LEICA DMi1 microscope (Leica Microsystems CMS GmbH, Wetzlar, Germany). The percentage of morphologically changed cells on the total amount of cells per picture was quantified using neuralab.de (Neuralab, Ulm, Germany) and Excel software (Microsoft Corporation, Redmond, WA, United States). Cell viability was analyzed via Cell Titer 96^®^ Aqueous One Solution Cell Proliferation Assay (MTS Assay; Promega GmbH, Walldorf, Germany) following the manufacturer’s instructions. Cells treated with 20% (v/v) dimethyl sulfoxide (DMSO; Carl Roth, Karlsruhe, Germany) served as control for reduced viability.

### 2.4 Purification and labeling of proteins

Recombinant expression, purification and eventually activation of C2I, C2IIa, PA_83_, PA_63_ and His-DTA is described elsewhere (Barth et al., 1998a; 2000; Wesche et al., 1998; Melnyk et al., 2006; Eisele et al., 2022; Heber et al., 2023). Unnicked DT from *C. diphtheriae* was purchased from Merck (Merck, Darmstadt, Germany). LF_N_-DTA was kindly provided by R. John Collier (Harvard Medical School, Boston, United States). To obtain C2I^405^ and C2IIa^488^, C2IIa and C2I (both solved in PBS) were labeled according to the manufacturer’s protocol with DyLight^®^ 488 NHS Ester and DyLight^®^ 405 NHS Ester (Thermo Fisher Scientific, Rockford IL, USA), respectively. Excessive dye was removed using Zeba™ Spin Desalting Columns (7K MWCO, Thermo Fisher Scientific, Waltham, MA, United States). In parallel, PBS without any protein was processed identical to C2I and C2IIa to obtain a solvent control for experiments performed with C2I^405^ and C2IIa^488^.

### 2.5 Flow cytometric binding assay

To detach the cells from culture dishes, they were incubated in 25 mM ethylenediaminetetraacetate (EDTA) in PBS (137 mM NaCl, 2.7 mM KCl, 8 mM Na_2_HPO_4_, 1.8 mM KH_2_PO_4_; pH 7.4) for 20 min (37°C) and rinsed gently. After three times washing with PBS (centrifugation at 500 rpm for 5 min, discard of supernatant) the cells were resuspended in PBS^++^ (PBS with 0.9 mM CaCl_2_ and 0.5 mM MgCl_2_). 2 × 10^5^ cells in 200 µL PBS^++^ per condition were pre-incubated (37°C, 30 min) with or without DSF and/or Cu (II) as indicated in the individual experiments. Afterwards, the cells were chilled on ice for 5 min, and the respective toxins (C2IIa^488^, C2I^405^, DT) or an identical volume of PBS (Ctrl) were added and incubation on ice was performed for 30 min to allow binding but no internalization. Note: For the PBS added to the Ctrl in Figures 2A–D (C2IIa^488^/C2I^405^), see also Section 2.4 (Protein Purification and Labelling). The cells in Figures 2A–D (C2IIa^488^/C2I^405^) were washed with ice cold PBS^++^ to remove unbound C2IIa^488^ and C2I^405^ and resuspended in 200 µL PBS^++^. The cells in Figure 8A and B were washed with ice cold PBS^++^ to remove unbound DT and incubated on ice for 30 min with a mouse anti-diphtheria toxin antibody (1:200 in 200 µL PBS^++^; 8G2, Bio-Rad Laboratories, Hercules, CA, United States), washed again with PBS^++^ and incubated on ice for 30 min with an Alexa Fluor™ 488 goat anti-mouse IgG (H + L) antibody (1:500 in 200 µL PBS^++^; Thermo Fisher Scientific, Waltham, MA, United States), then they were washed again in PBS^++^ and finally resuspended in 200 µL PBS^++^. Fluorescence was measured using a BD FACS Celesta™ flow cytometer (Becton, Dickinson and Company, Franklin Lakes, NJ, United States) and BD FACSDiva™ software 8.0.1.1. Data were analyzed via Flowing Software v2.5.1. (Turku Centre of Biotechnology, Finland).

### 2.6 Lipid bilayer membrane assay

Lipid Bilayer Membrane Assays were conducted as indicated in the individual experiments using a Nanion Orbit mini system (Nanion Technologies GmbH, Munich, Germany). Planar lipid bilayers were formed over cavities with a diameter of 100 µm on a micro cavity array chip (MECA4, IonEra GmbH, Freiburg, Germany) as described earlier (Baaken et al., 2008) by using 10 mg/mL 1,2-diphytanoyl-sn-glycero-3-phosphocholine (Avanti Polar Lipids, Alabaster, United States) dissolved in n-octane (Sigma-Aldrich, Steinheim, Germany). Pore formation was induced by addition of the pore-forming proteins to the *cis*-side of the membrane and the conduction-blocking potential of DSF was elucidated as indicated.

### 2.7 *In-vitro* enzyme activity assay

In a total volume of 25 µL per condition, 30 µg HeLa cell lysate in either ADP-ribosylation buffer (20 mM Tris-HCl, 1 mM EDTA, 1 mM dithiothreitol (DTT), 5 mM MgCl_2_, pH 7.5) supplemented with Complete™ protease inhibitor (1:50 diluted; Merck, Darmstadt, Germany) or LF cleavage buffer (25 mM KH_2_PO4, 20 mM NaCl, 10 mM MgCl_2_, 1 mM DTT, 0.1 mM CaCl_2_, 0.1 mM ZnCl_2_, 0.1 mM ADP, 10% glycerol (v/v)) (Vitale et al., 2000) were incubated for 30–60 min (see individual experiment) with indicated concentrations of LF, C2I and His_DTA with or without DSF (see individual experiments), and in the case of C2I and His_DTA with 250 pmol biotin-labelled NAD^+^. Then, the reaction was stopped by addition of Laemmli buffer (0.3 M Tris-HCl, 10% SDS, 37.5% glycerol, 0.4 mM bromophenol blue, 100 mM DTT) and heating at 95°C for 10 min. Afterwards, the samples were transferred to SDS-PAGE followed by Western Blot and the amount of modified (e.g., biotin-ADP-ribosylated or cleaved) substrate was detected via streptavidin-peroxidase conjugate or MEK-1 N-Terminus antibody, respectively.

### 2.8 SDS-PAGE and Western blot

Electrophoresis was performed using 12.5% acrylamide gels. Following protein separation, gels were either stained with Coomassie Brilliant Blue R250 (SERVA Electrophoresis GmbH, Heidelberg, Germany) or proteins were transferred onto nitrocellulose membranes via semi-dry Western Blotting technique. Protein transfer was validated by Ponceau S (AppliChem GmbH, Darmstadt, Germany) and membranes were blocked with 5% skim milk powder in PBS-T (PBS with 0.1% Tween20 (Merck, Darmstadt, Germany)) for 30 min at room temperature (RT). Afterwards, membranes were incubated for 1 h at RT with either streptavidin-peroxidase conjugate or the indicated primary antibodies diluted in PBS-T. Streptavidin-peroxidase conjugate (Sigma-Aldrich, St. Louis, MO, United States) was diluted 1:5000 in PBS-T and mouse MEK-1 N-Terminus antibody (nanoTools, Teningen, Germany) was diluted 1:500 in PBS-T. Loading controls (GAPDH and Hsp90) were detected by 1:2000 diluted mouse GAPDH antibody (G-9, Santa Cruz Biotechnology, Dallas, TX, United States) or 1:1000 diluted mouse Hsp90 α/β antibody (F-8, Santa Cruz Biotechnology, Dallas, TX, United States), respectively. Following three times washing with PBS-T, membranes were incubated for 1 h at RT with the respective secondary antibodies (1:2500 diluted in PBS-T) Goat anti-Mouse IgG (H + L) secondary antibody, horse-reddish peroxidase (HRP) (Thermo Fisher Scientific, Waltham, MA, United States), or mouse anti-rabbit IgG-HRP (Santa Cruz Biotechnology, Dallas, TX, United States). Unbound antibodies were removed by three times washing with PBS-T. HRP-conjugated antibodies or streptavidin were detected with Pierce ECL Western blotting substrate (Thermo Fisher Scientific, Waltham, MA, United States) and X-ray films (AGFA Healthcare, Mortsel, Belgium).

### 2.9 Membrane translocation assay

In order to mimic and examine the pH-dependent translocation of the investigated toxins across the endosomal membrane, a well-established Membrane Translocation Assay (Sandvig and Olsnes, 1980; Kreidler et al., 2017) was performed as described earlier (Heber et al., 2023). In brief, the physiological cytosolic translocation of the toxins’ enzymatic subunits across the membranes of acidified endosomes was inhibited by bafilomycin A1 (100 nM), which blocks endosomal acidification. The toxins were allowed to bind to the cells at 4°C for 30 min and unbound toxins were removed by washing. Afterwards, the cells were treated for 10 min at 37°C either with an acidic medium (pH 3.8) to induce direct translocation of the enzymatic subunits across the cell membrane, or with neural medium as control. Then, the medium was exchanged to serum-containing medium containing 100 nM bafilomycin A1 and the cells were analyzed as indicated. Where indicated, DSF in a concentration of 15 µM was present during the whole experiment.

### 2.10 Statistics

If not stated otherwise, all experiments were performed at least three times independently. Statistical analysis was performed with individual replicates (n) as indicated in the respective experiments. *p* values were defined as follows: not significant (ns) *p* ≥ 0.05, *p < 0.05, **p < 0.01, ***p < 0.001, ****p < 0.0001.

## 3 Results

### 3.1 DSF protects HeLa cells from C2 toxin-mediated intoxication

**FIGURE 1:**
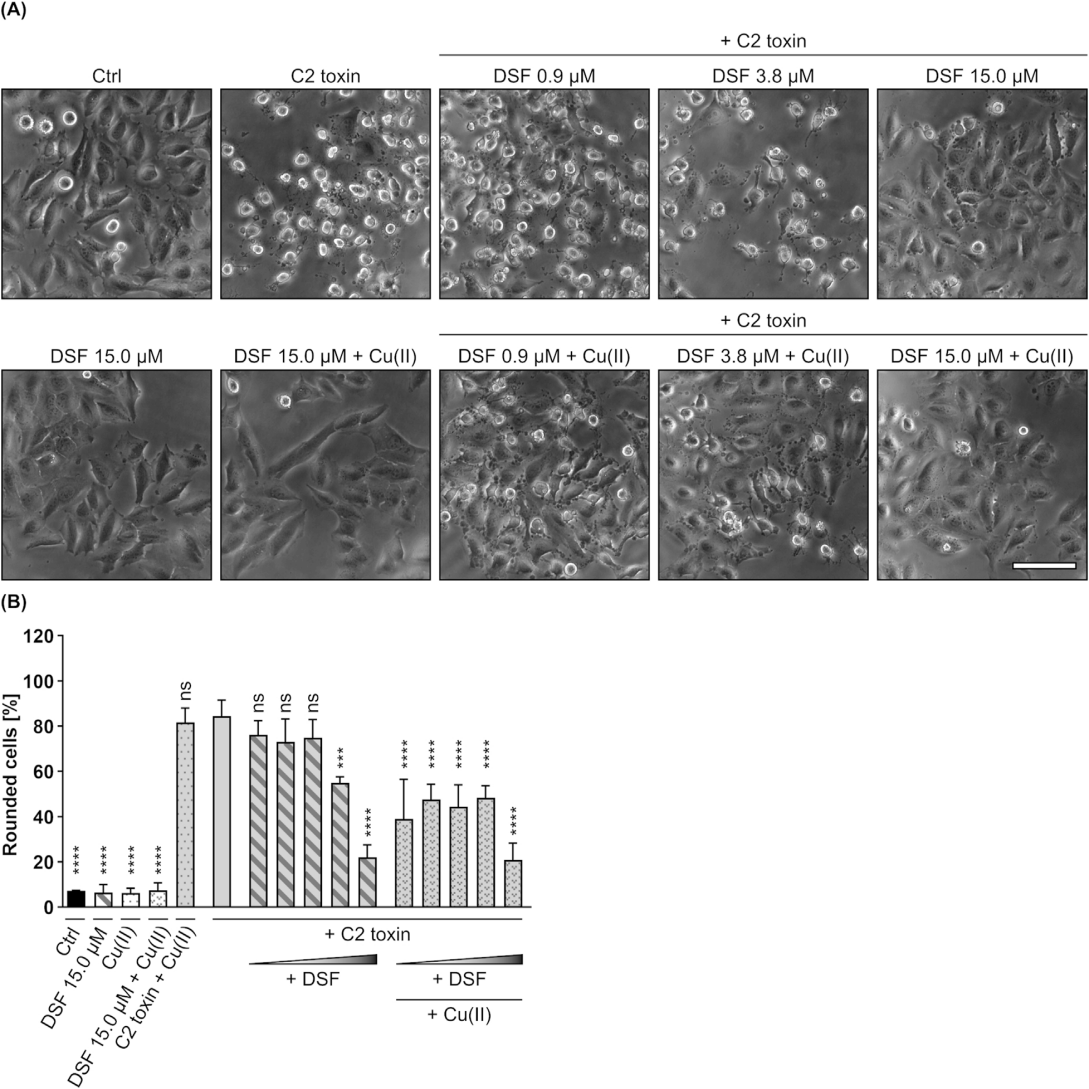
DSF prevents HeLa cells from C2 toxin-induced morphological changes. **(A)** Representative pictures after 5 h incubation time under the indicated conditions. C2 toxin concentration was 60 ng/mL (≙ 1.21 nM) C2I with 120 ng/mL (≙ 2.01 nM) C2IIa. Cu(II) D-Gluconate (Cu(II)) concentration was 330 nM. Sale bar corresponds to 100 µm. **(B)** Quantitative evaluation of cell rounding after 5 h incubation with 0.94 µM, 1.88 µM, 3.75 µM, 7.50 µM or 15 µM DSF, 330 nM Cu(II) and C2 toxin (60 ng/mL C2I + 120 ng/mL C2IIa) in the indicated combinations, or without any compound as control (Ctrl). Values are given as mean ± SD (n = 3) of triplicates from one representative experiment of three independent replicates. Statistical analysis was performed and conditions were compared to the C2 toxin only condition by using one-way ANOVA with Dunnett’s correction for multiple comparison (ns *p* ≥ 0.05, ***p < 0.001, ****p < 0.0001).

To evaluate whether DSF inhibits C2 toxin, HeLa cells were incubated for 5 h with C2 toxin in the presence or absence of increasing DSF concentrations (0.94 µM–15 µM), that have previously been proven to show no adverse effects on HeLa cell viability (Supplementary Figures 1A, B) and lie within the range of DSF plasma concentrations in patients treated for alcoholism (up to 20 µM) (Beaudry et al., 2023). Additional cells were either treated with the highest concentration of DSF, or were left untreated as control (Ctrl). As depicted in Figures 1A,B, cells treated with C2 toxin showed typical toxin-induced rounding, which was progressively reduced in the presence of increasing DSF concentrations. This indicates a concentration-dependent inhibition of C2 toxin by DSF. Moreover, it was demonstrated previously that DSF shows enhanced activity in the presence of copper. It was therefore tested whether the inhibitory potential of DSF can be further improved when copper gluconate (Cu(II)) is co-applied, which indeed was the case (Figures 1A,B). While Cu(II) alone or in combination with DSF had no effect on HeLa cells after 5 h, their combination led to an enhanced inhibition of C2 toxin. When applied together with Cu(II), an 8-fold lower concentration of DSF (0.94 µM) showed comparable inhibitory activity towards C2 toxin as 7.5 µM DFS without Cu(II). Since at the same time Cu(II) alone had no effect at all on the intoxication of HeLa cells with C2 toxin, these results indicate an enhancement of the DSF-dependent inhibition by Cu(II) rather than an additive inhibitory effect of both components. Potential inhibitory effects of the DSF solvent EtOH in combination with or without Cu(II) were excluded (Supplementary Figures 2A, B).

### 3.2 DSF has no effect on the binding of C2IIa and C2I, but affects the enzyme activity of C2I and interferes with its translocation by a potential blockage of the C2IIa pore

**FIGURE 2:**
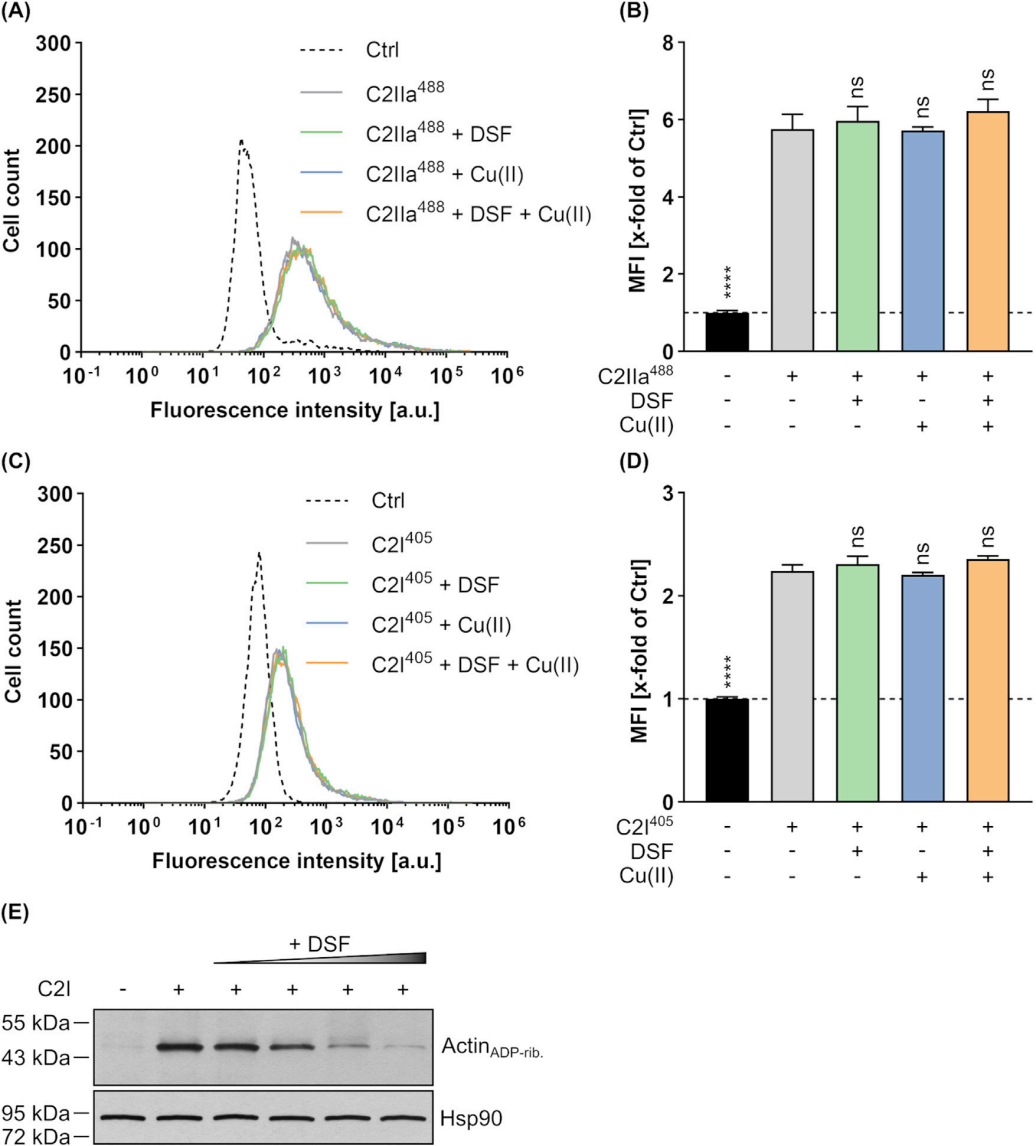
Effect of DSF and Cu(II) on the cellular binding and enzyme activity of C2 toxin. To simultaneously analyze the binding of C2IIa (800 ng/mL) and C2I (800 ng/mL) to HeLa cells in the presence or absence of 15 µM DSF and 330 nM Cu(II), flow cytometric analysis was performed with fluorescently labelled proteins. Control (Ctrl) cells were treated with PBS. The data depicted in **(A–D)** were generated within the same experiment by using different labels (excitation at 405 nm or 488 nm) for C2I and C2IIa and measurement of both signals at the same time. **(A)** Representative histogram with fluorescence intensity values for C2IIa^488^. **(B)** The median fluorescence intensity (MFI) values for C2IIa^488^ of all samples were measured and normalized to the Ctrl. **(C)** Representative histogram with fluorescence intensity values for C2I^405^. **(D)** The MFI values for C2I^405^ of all samples were measured and normalized to the Ctrl. Values are given as mean ± SD (n = 2) of duplicates from one representative experiment of three independent replicates. Statistical analysis was performed and conditions were compared to the C2 toxin only condition by using one-way ANOVA with Dunnett’s correction for multiple comparison (ns *p* ≥ 0.05, ****p < 0.0001). **(E)** Representative Western Blot of an *in vitro* enzyme activity assay. HeLa lysate was incubated (30 min, 37°C) with biotinylated NAD^+^ and 1 nM C2I in the presence or absence of 15 μM, 30 μM, 60 µM or 120 µM DSF. As control, lysate was incubated without C2I or DSF. ADP-ribosylated, i.e., biotinylated Actin (Actin_ADP-rib._) was detected using a streptavidin-peroxidase conjugate. Hsp90 was detected as control for equal protein loading.

To investigate the underlying mechanism behind the concentration-dependent inhibition of C2 toxin by DSF, potential effects of DFS on binding, enzyme activity and translocation of C2 toxin were investigated. First, possible effects of DSF on the binding of C2IIa to cells and C2I were investigated by flow cytometry-based binding experiments. To enable simultaneous analysis of C2IIa and C2I, both components were labelled with different fluorescent dyes (C2IIa with green fluorescent DyLight^®^ 488 NHS Ester [C2IIa^488^] and C2I with blue fluorescent DyLight^®^ 405 NHS Ester [C2I^405^]) and incubated with HeLa cells on ice (allows binding but not uptake) in the presence or absence of DSF and/or Cu(II). Moreover, PBS treated cells served as control and were used for normalization of the measured fluorescent signals. Following removal of unbound C2IIa^488^ and C2I^405^ by washing, the cells were analyzed via Flow Cytometry (Figures 2A–D). Here, fluorescence intensities of individual cells correlate with the amount of bound C2IIa^488^ or C2I^405^, respectively. As shown, neither DSF, nor Cu(II) or the combination of both had any effect on the binding capacity of C2IIa^488^ (Figures 2A,B) or C2I^405^ (Figures 2C,D). Thus, the inhibition of C2 toxin by DSF seems not to be caused by interference of DSF with the binding of C2IIa or C2I.

Besides effects on C2 toxin binding, a potential impact of DSF on the enzyme component C2I was tested in an *in vitro* enzyme activity assay. To this end, HeLa cell lysate was incubated with or without C2I, increasing concentrations of DSF and biotin-labelled NAD^+^. Subsequently, the level of ADP-ribosylated and thus biotinylated Actin (Actin_ADP-rib._) was detected via Western Blot, whereby strong signals indicate high and weak signals indicate reduced enzyme activity, respectively. As depicted in Figure 2E, increasing concentrations of DSF led to decreasing Actin_ADP-rib._ signals, indicating a concentration-dependent inhibition of C2I activity by DSF. It is, however, noteworthy to point out that only higher concentrations of DSF (>15 µM) than used in cell-based assays (Figures 1A,B) showed a strong inhibition of C2I and that such DSF concentrations showed adverse effects on HeLa cell viability in the absence of any toxin (Supplementary Figures 1A, B).

**FIGURE 3:**
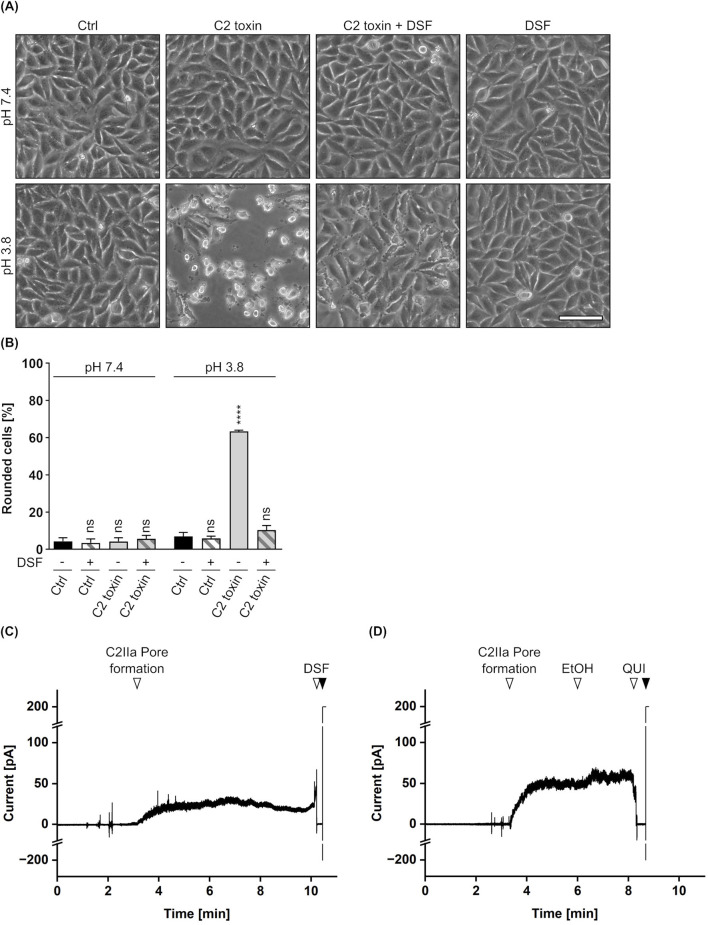
Effect of DSF on C2IIa-mediated translocation and conductance. **(A)** Assay for translocation across the plasma membrane via acidic pulse. HeLa cells were treated according to the assay protocol with C2 toxin (100 ng/mL C2I + 200 ng/mL C2IIa) and 15 µM DSF in the indicated combinations, or without any compound as control (Ctrl). Pictures were taken 5 h after the acidic pulse and representative pictures are depicted. The upper panel shows cells pulsed with medium at pH 7.4, while the lower panel shows cells pulsed with medium at pH 3.8. Scale bar corresponds to 100 µm. **(B)** Quantification of the assay for translocation from **(A)**. Shown is the percentage of rounded cells 5 h after the acidic pulse. Values are given as mean ± SD (n = 2) of duplicates from one representative experiment of three independent replicates. Statistical analysis was performed compared to the respective Ctrl at pH 7.4 or pH 3.8 by using one-way ANOVA with Dunnett’s correction for multiple comparison (ns *p* ≥ 0.05, ****p < 0.0001). **(C)** DSF blocks C2IIa pores in lipid bilayer membrane experiments *in vitro*. The aqueous phase consisted of an electrolyte solution (200 mM KCl, 10 mM HEPES, pH 6.0). A lipid bilayer membrane consisting of 1,2-diphytanoyl-sn-glycero-3-phosphocholine solved in n-octane was formed, which acts isolating and results in zero level of conductance (starting point). Addition of 1000 ng/mL C2IIa to the *cis*-side of the membrane resulted in C2IIa pore-induced conductance (▽). Subsequent addition of 30 µM DSF (▽) immediately blocked any current from flowing, resulting in zero level of conductance. Finally, disruption (▼) of the lipid bilayer membrane via an electric pulse resulted in maximum conductance (200 pA) and confirmed the former existence of an intact, isolating lipid bilayer membrane. Temperature was constant at 25°C, and the applied voltage was 20 mV. **(D)** Control experiment for **(C)** under identical conditions but with the addition of a volume (0.3% (v/v)) of ethanol (EtOH) equal to DSF (solvent control) and 30 µM quinacrine (QUI) as an established C2IIa pore blocker.

Finally, potential effects of DSF on the translocation of C2I through the C2IIa pore were elucidated in a membrane translocation assay (Sandvig and Olsnes, 1980; Kreidler et al., 2017; Heber et al., 2023). In this assay, the pH-driven translocation of cell-bound C2 toxin across the plasma membrane is triggered by an acidic pulse, mimicking the prevailing conditions in acidified endosomes during toxin uptake into cells. Importantly, endosomal acidification via vacuolar-type-ATPases was inhibited by bafilomycin A1 during the whole experiment, excluding intoxication of the cells via the endosomal pathway. As shown in Figures 3A,B, HeLa cells treated with C2 toxin showed typical C2 toxin-mediated rounding 5 h after the acidic pulse. This phenotype (i.e., intoxication) was completely prevented in the presence of DSF and a cell morphology similar to untreated control cells or cells treated with DSF only was maintained. This strongly suggest an interference of DSF with the translocation of C2I through the C2IIa pore. With respect to these observations, potential effects of DSF on the C2IIa translocation pore were investigated in closer detail. To this end, the C2IIa pore-mediated ion current through planar lipid bilayer membranes, which depends on the number and the single-channel conductivity of the incorporated C2IIa pores, was measured before and after the addition of DSF, its solvent ethanol (EtOH) or the established C2IIa pore blocker quinacrine (QUI) (Schmid et al., 1994; Neumeyer et al., 2008; Kreidler et al., 2017). As depicted in Figures 3C,D, formation of C2IIa pores in an insulating lipid bilayer membrane facilitated a measurable ion current. While addition of the solvent EtOH showed no inhibition of the current flow at all (Figure 3D), an addition of DSF led to an immediate block of any current flowing and resulted in zero measurable conductance, similar to the situation previous to pore formation (Figure 3C). Interestingly, this abrupt and complete blockage of conductance after the addition of DSF was similar to the picture received when the established C2IIa pore blocker QUI was added to C2IIa pores under identical conditions (Figure 3D). These results strongly indicate a C2IIa pore-blocking activity of DSF as a potential mode of inhibition of C2 toxin.

### 3.3 The combination of DSF and Cu(II) protects J774A.1 cells from LT-mediated toxicity

**FIGURE 4:**
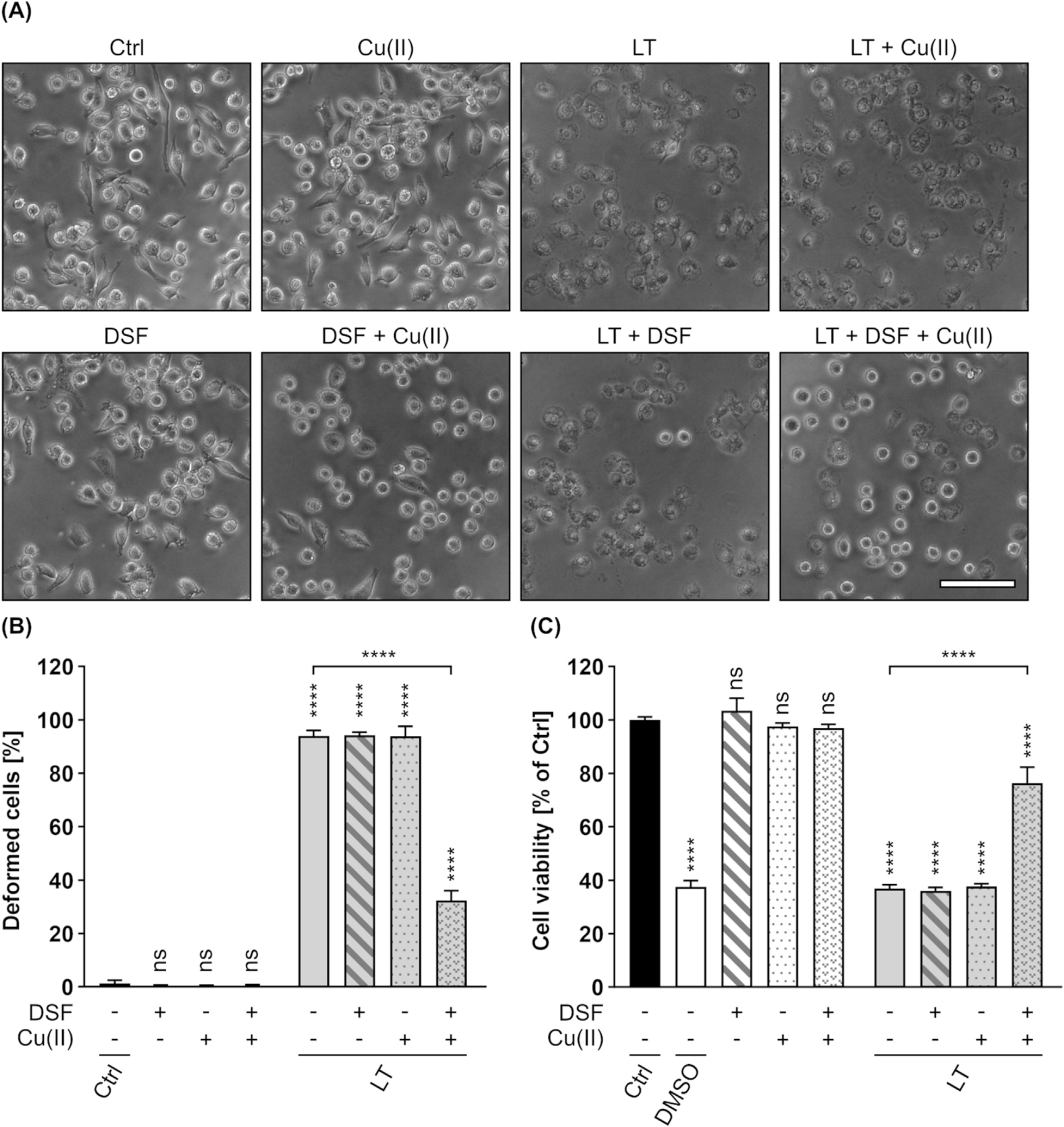
DSF in combination with Cu(II) prevents J774A.1 cells from toxic effects on their morphology and cell viability caused by Lethal toxin (LT). **(A)** Representative pictures after 5 h incubation time under the indicated conditions. LT concentration was 2.4 nM PA_83_ with 2.2 nM LF. Cu(II) concentration was 330 nM. Sale bar corresponds to 100 µm. **(B)** Quantitative evaluation of cell rounding after 5 h incubation with 15 µM DSF, 330 nM Cu(II) and LT (2.4 nM PA_83_ + 2.2 nM LF) in the indicated combinations, or without any compound as control (Ctrl). Values are given as mean ± SD (n = 3) of triplicates from one representative experiment of three independent replicates. Statistical analysis was performed and all conditions were compared to the Ctrl condition by using one-way ANOVA with Dunnett’s correction for multiple comparison (ns *p* ≥ 0.05, ****p < 0.0001). Additional statistical analysis was performed as indicated by the black connection line using Šídák’s multiple comparison test (****p < 0.0001). **(C)** Relative viability (% of Ctrl) of the cells from **(B)** measured via MTS assay. As an additional control for reduced viability, cells were incubated with toxic concentrations (20%) of DMSO. Values are given as mean ± SD (n = 3) of triplicates from one representative experiment of three independent replicates. Statistical analysis was performed and all conditions were compared to the Ctrl condition by using one-way ANOVA with Dunnett’s correction for multiple comparison (ns *p* ≥ 0.05, ****p < 0.0001). Additional statistical analysis was performed as indicated by black brackets using one-way ANOVA with Šídák’s multiple comparison test (****p < 0.0001).

Since the binding and transport components C2II (C2 toxin) and PA (anthrax toxin) are known to share high homology in sequence and structure (Schleberger et al., 2006; Neumeyer et al., 2008), it was assumed that the inhibitory effects of DSF shown for the C2IIa pore might be transferrable to PA. In a first step, it was therefore tested whether DSF is able to inhibit the LT-mediated intoxication of J774A.1 cells, a murine macrophage-like cell line known to react on LT intoxication by a change in morphology and reduced cell viability (Lin et al., 1996; Bhatnagar et al., 1999; Kreidler et al., 2017). An intoxication assay was performed by incubating J774A.1 cells for 5 h with LT in the presence or absence of DSF and/or Cu(II). Further cells were incubated with DSF and/or Cu(II), or without any compound as control. Noteworthy, it was shown in preliminary experiments, that J774A.1 cells are more susceptible towards DSF and show adverse effects on cell viability at lower DSF concentrations than HeLa cells (Supplementary Figures 1A–D). The concentration of DSF used in this experiment was therefore reduced to 3.75 µM, a concentration that did not show inhibition of C2 toxin on HeLa cells in the absence of Cu(II) (Figures 1A,B). Comparable to the findings for C2 toxin, 3.75 µM DSF alone did not show any inhibitory activity towards LT (Figures 4A–C). In contrast to that, the combination of 3.75 µM DSF and Cu(II) reduced the portion of morphologically changed cells and the loss in cell viability by more than half compared to cells treated with LT only (Figures 4A–C). Since neither DSF nor Cu(II) alone showed any inhibition of LT, these results strongly imply an enhanced inhibitory activity towards LT for the combination of DSF and Cu(II).

### 3.4 DSF inhibits the intoxication of HeLa cells by PA_83_ with LF_N_-DTA or His-DTA

**FIGURE 5:**
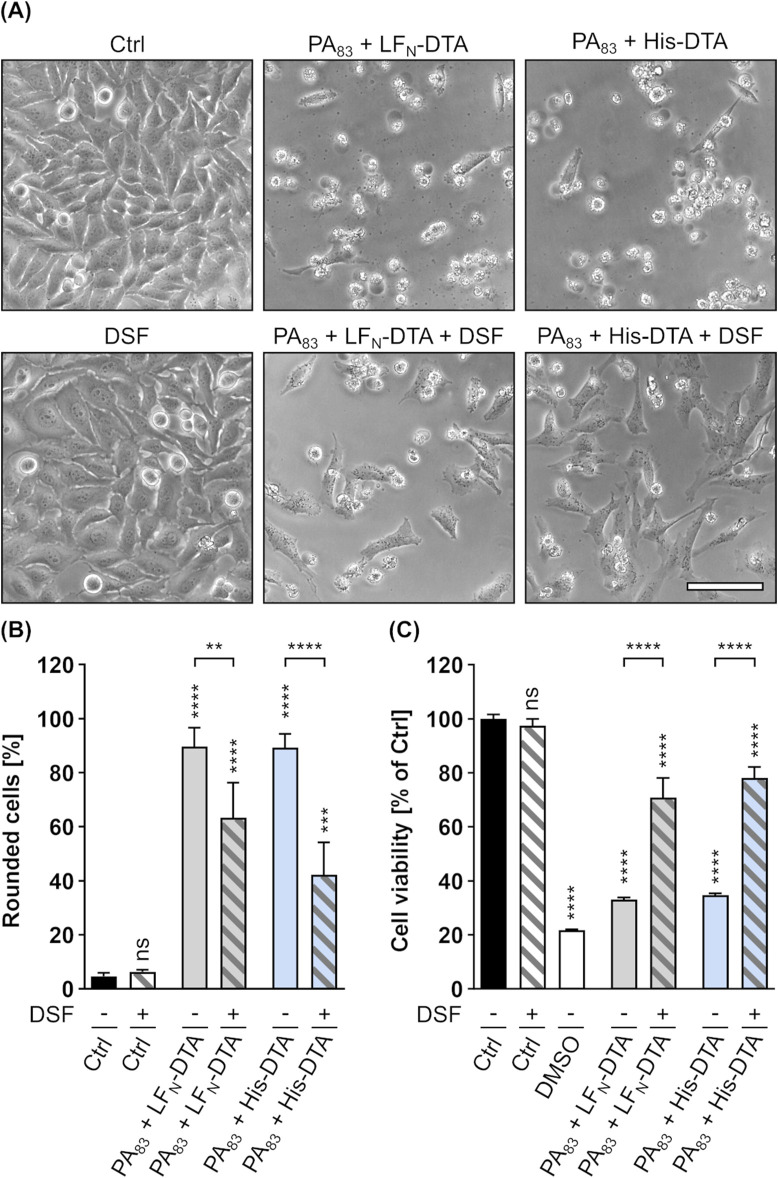
DSF prevents HeLa cells from toxic effects on their morphology and cell viability caused by a combination of PA_83_ with His-DTA or LF_N_-DTA. **(A)** Representative pictures after 24 h incubation time under the indicated conditions. Toxin concentrations were 0.3 nM PA_83_ with 0.28 nM LF_N_-DTA and 2.5 nM PA_83_ with 25 nM His-DTA. Sale bar corresponds to 100 µm. **(B)** Quantitative evaluation of cell rounding after 24 h incubation with 15 µM DSF and 0.3 nM PA_83_ with 0.28 nM LF_N_-DTA or 2.5 nM PA_83_ with 25 nM His-DTA in the indicated combinations, or without any compound as control (Ctrl (−)). Values are given as mean ± SD (n = 3) of triplicates from one representative experiment of three independent replicates. Statistical analysis was performed and all conditions were compared to the Ctrl (−) condition by using one-way ANOVA with Dunnett’s correction for multiple comparison (ns *p* ≥ 0.05, ***p < 0.001, ****p < 0.0001). Additional statistical analysis was performed as indicated by black brackets using Šídák’s multiple comparison test (**p < 0.01, ****p < 0.0001). **(C)** Relative viability (% of Ctrl (−)) of the cells from **(B)** measured via MTS assay. As an additional control for reduced viability, cells were incubated with toxic concentrations (20%) of DMSO. Values are given as mean ± SD (n = 3) of triplicates from one representative experiment of three independent replicates. Statistical analysis was performed and all conditions were compared to the Ctrl (−) condition by using one-way ANOVA with Dunnett’s correction for multiple comparison (ns *p* ≥ 0.05, ****p < 0.0001). Additional statistical analysis was performed as indicated by black brackets using one-way ANOVA with Šídák’s multiple comparison test (****p < 0.0001).

In order to examine whether higher concentrations of DSF can inhibit the PA_83_-dependent intoxication of cells without Cu(II), PA_83_ and DSF were tested in a HeLa-based setup that allows the use of identical DSF concentrations as for C2 toxin (see Figures 1A,B). To elucidate PA_83_-mediated intoxication of HeLa cells, two well-established fusion toxin systems were used in which PA_83_ delivers LF_N_-DTA or His-DTA into the cytosol of HeLa cells. LF_N_-DTA consists of the cell-impermeable, enzymatic domain of DT (DTA) (Pappenheimer, 1977) that is fused to the non-toxic, N-terminal part of LF (LF_N_) serving as an adapter for the interaction and transport with PA_83_ (Arora and Leppla, 1994; Rabideau and Pentelute, 2016). The N-terminally His-tagged His-DTA on the other hand has been shown previously to be transported into cells via PA_83_, most probably by an electrostatic interaction between the positively charged His-tag and the negatively charged PA pore lumen (Blanke et al., 1996; Collier, 2009; Heber et al., 2023). The PA_83_-mediated uptake of LF_N_-DTA or His-DTA results in cell rounding and reduced cell viability, which is characteristic for DT-intoxication (Strauss and Hendee, 1959; Heber et al., 2023). As shown in Figures 5A–C, 24 h treatment of HeLa cells with PA_83_ and LF_N_-DTA or His-DTA led to typical cell rounding and a reduction in cell viability. However, the presence of 15 µM DSF could significantly reduce the amount of rounded cells and the loss in cell viability. Treatment of HeLa cells with an identical concentration (15 µM) of DSF without any toxin showed no differences to untreated control cells (Figures 5A–C). These observations strongly suggest a protective activity of DSF towards HeLa cell intoxication by PA_83_/LF_N_-DTA and PA_83_/His-DTA. The finding, that DSF inhibits the PA_83_-dependent intoxication of HeLa cells in the absence of Cu(II) in this setup indicates that the observed inhibition of LT by DSF and Cu(II) (see Figures 4A–C) is rather driven by DSF than by Cu(II). Moreover, this suggestion is further strengthened by the finding that the DSF solvent EtOH neither alone nor in combination with Cu(II) had any effect on the PA_83_-dependent intoxication of HeLa cells (Supplementary Figures 2C, D).

### 3.5 DSF has no effect on the enzyme activity of LF or DTA, but interferes with PA_83_ pore-mediated translocation and conductance

**FIGURE 6:**
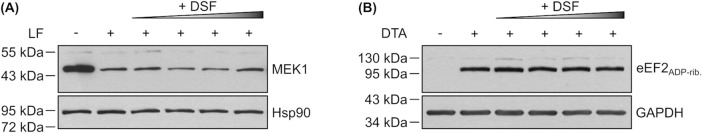
Effect of DSF on the enzyme activity of LF and DTA. **(A)** Representative Western Blot of an *in vitro* enzyme activity assay. HeLa lysate was incubated (45 min, 37°C) with 5 nM LF in the presence or absence of 15 μM, 30 μM, 60 µM or 120 µM DSF. As control, lysate was incubated without LF or DSF. Non-cleaved MEK1 was detected using an antibody against its N-Terminus, which is cleaved by LF. Hsp90 was detected as control for equal protein loading. **(B)** Representative Western Blot of an *in vitro* enzyme activity assay. HeLa lysate was incubated (30 min, 37°C) with biotinylated NAD^+^ and 200 nM DTA in the presence or absence of 15 μM, 30 μM, 60 µM or 120 µM DSF. As control, lysate was incubated without DTA or DSF. Biotinylated, i.e., ADP-ribosylated eukaryotic elongation factor 2 (eEF2_ADP-rib._) was detected using a streptavidin-peroxidase conjugate. GAPDH was detected as control for equal protein loading.

Following the observation that DSF is able to inhibit the intoxication of cells by PA_83_/LT, PA_83_/LF_N_-DTA or PA_83_/His-DTA, the mechanistic effects by which DSF was shown to inhibit C2 toxin were elucidated in closer detail for PA, LF and DTA too. First, potential effects of DSF on the enzyme activity of LF or DTA were analyzed *in vitro*. To this end, HeLa lysate was incubated with increasing concentrations of DSF in the presence or absence of LF or DTA. In case of DTA, biotin-labelled NAD^+^ was also present to allow for quantification of ADP-ribosylated (i.e., biotinylated) eEF2 (eEF2_ADP-rib._). The respective levels of cleaved MEK1 (LF activity) or eEF2_ADP-rib._ (DTA activity) were then analyzed via Western Blot. As shown in Figures 6A,B, none of the used DSF concentrations had any effect on the enzyme activities of LF or DTA, as band intensities are identical for the conditions with toxins only or toxins together with DSF, respectively. These results suggest, that the inhibitory effect of DSF towards the intoxication of cells with combinations of PA_83_ and LF, LF_N_-DTA or His-DTA is not based on an inhibition of the respective catalytic activity of the toxins.

**FIGURE 7:**
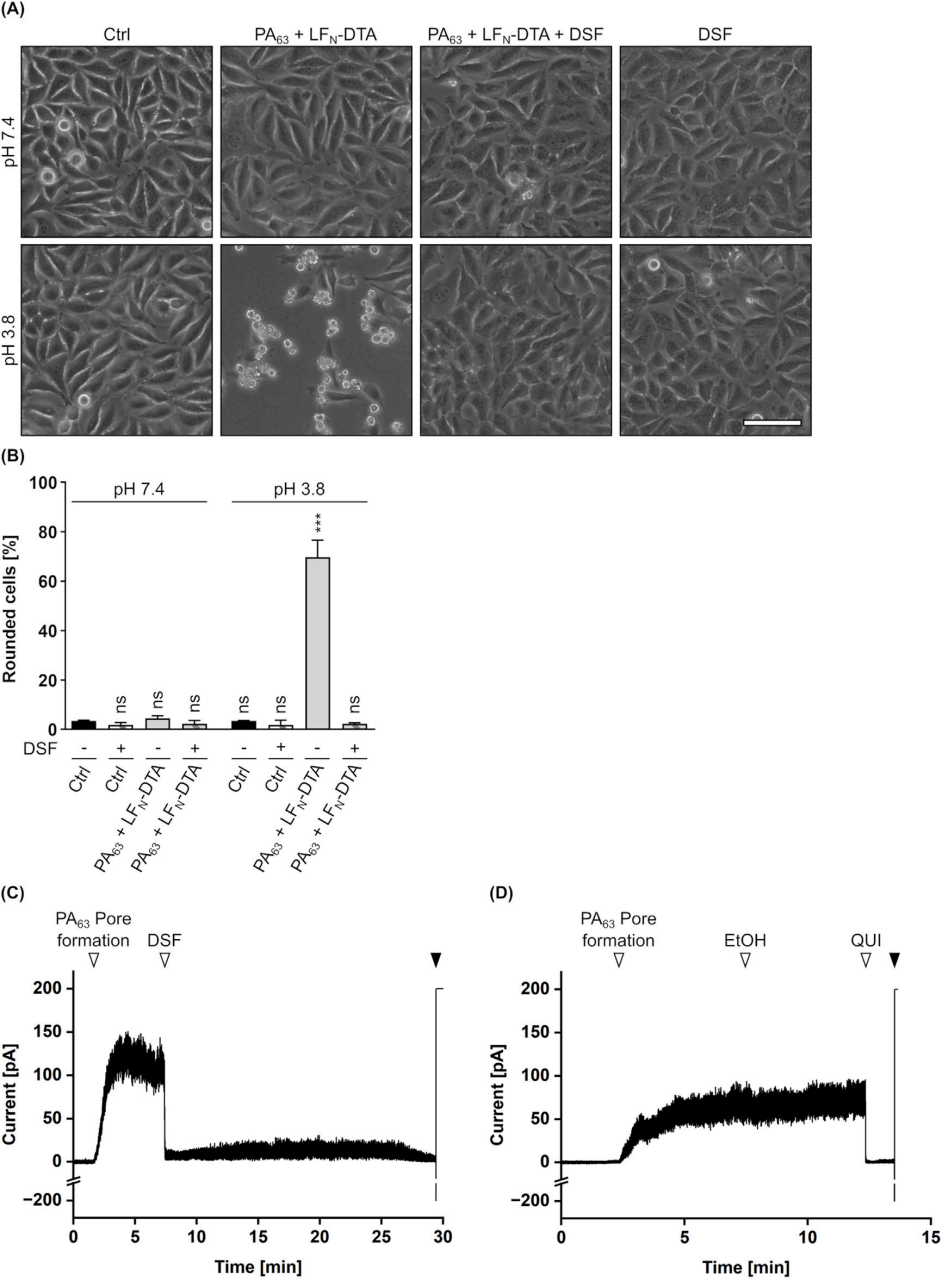
Effect of DSF on PA_63_-mediated translocation and conductance. **(A)** Assay for translocation across the plasma membrane via acidic pulse. HeLa cells were treated according to the assay protocol with 24 nM PA_63_ + 30 nM LF_N_-DTA and 15 µM DSF in the indicated combinations, or without any compound as control (Ctrl). Pictures were taken 5 h after the acidic pulse and representative pictures are depicted. The upper panel shows cells pulsed with medium at pH 7.4, while the lower panel shows cells pulsed with medium at pH 3.8. Scale bar corresponds to 100 µm. **(B)** Quantification of the assay for translocation from **(A)**. Shown is the percentage of rounded cells 5 h after the acidic pulse. Values are given as mean ± SD (n = 2) of duplicates from one representative experiment of three independent replicates. Statistical analysis was performed compared to the respective Ctrl (−) at pH 7.4 or pH 3.8 by using one-way ANOVA with Dunnett’s correction for multiple comparison (ns *p* ≥ 0.05, ***p < 0.001). **(C)** DSF blocks PA_63_ pores in lipid bilayer membrane experiments *in vitro*. The aqueous phase consisted of an electrolyte solution (150 mM KCl, 10 mM MES, pH 6.0). A lipid bilayer membrane consisting of 1,2-diphytanoyl-sn-glycero-3-phosphocholine solved in n-octane was formed, which acts isolating and results in zero level of conductance (starting point). Addition of 100 ng/mL PA_63_ to the *cis*-side of the membrane resulted in PA_63_ pore-induced conductance (▽). Subsequent addition of 30 µM DSF (▽) immediately blocked any current from flowing, resulting in zero level of conductance. Finally, disruption (▼) of the lipid bilayer membrane via an electric pulse resulted in maximum conductance (200 pA) and confirmed the former existence of an intact, isolating lipid bilayer membrane. Temperature was constant at 25°C, and the applied voltage was 20 mV. **(D)** Control experiment for **(C)** under identical conditions but with the addition of a volume (0.3% (v/v)) of ethanol (EtOH) equal to DSF (solvent control) and 30 µM Quinacrine (QUI) as an established PA_63_ pore blocker.

Besides enzyme activity, the translocation of LF_N_-DTA through PA_63_ pores was elucidated in a membrane translocation assay similar as described above (Section 3.2). In this assay, cells treated with PA_63_ and LF_N_-DTA clearly showed cell rounding (i.e., intoxication) 5 h after acidification, while cells treated with identical toxin concentrations showed no signs of intoxication when DSF was present (Figures 7A,B). For the combination of PA_63_/LF_N_-DTA with DSF, a phenotype comparable to untreated or DSF treated cells was maintained. This strongly indicates an interference of DSF with the translocation process of LF_N_-DTA through PA_63_ pores. Consequently, potential effects of DSF on the PA_63_ translocation pore were elucidated in a planar lipid bilayer assay as it was done before for the C2IIa pore (Section 3.2). As depicted in Figures 7C,D, the conductance facilitated by PA_63_ pores was analyzed when DSF, its solvent EtOH or the established PA_63_ pore blocker QUI (Orlik et al., 2005; Kreidler et al., 2017) were added. PA_63_ pore formation in an insulating lipid bilayer membrane induced a measurable ion current, that was not influenced at all by the addition of EtOH (Figures 7C,D). In contrast to that, addition of DSF immediately blocked any current flow, leading to zero level of conductance. Notably, this result is comparable to the outcome obtained when the established PA_63_ pore blocker QUI is added (Figures 7C,D). With respect to these results, it appears that that the inhibition of PA_63_-mediated cytotoxicity might derive from a PA_63_ pore-blocking activity of DSF.

### 3.6 DSF protects HeLa cells from DT-mediated toxic effects

**FIGURE 8:**
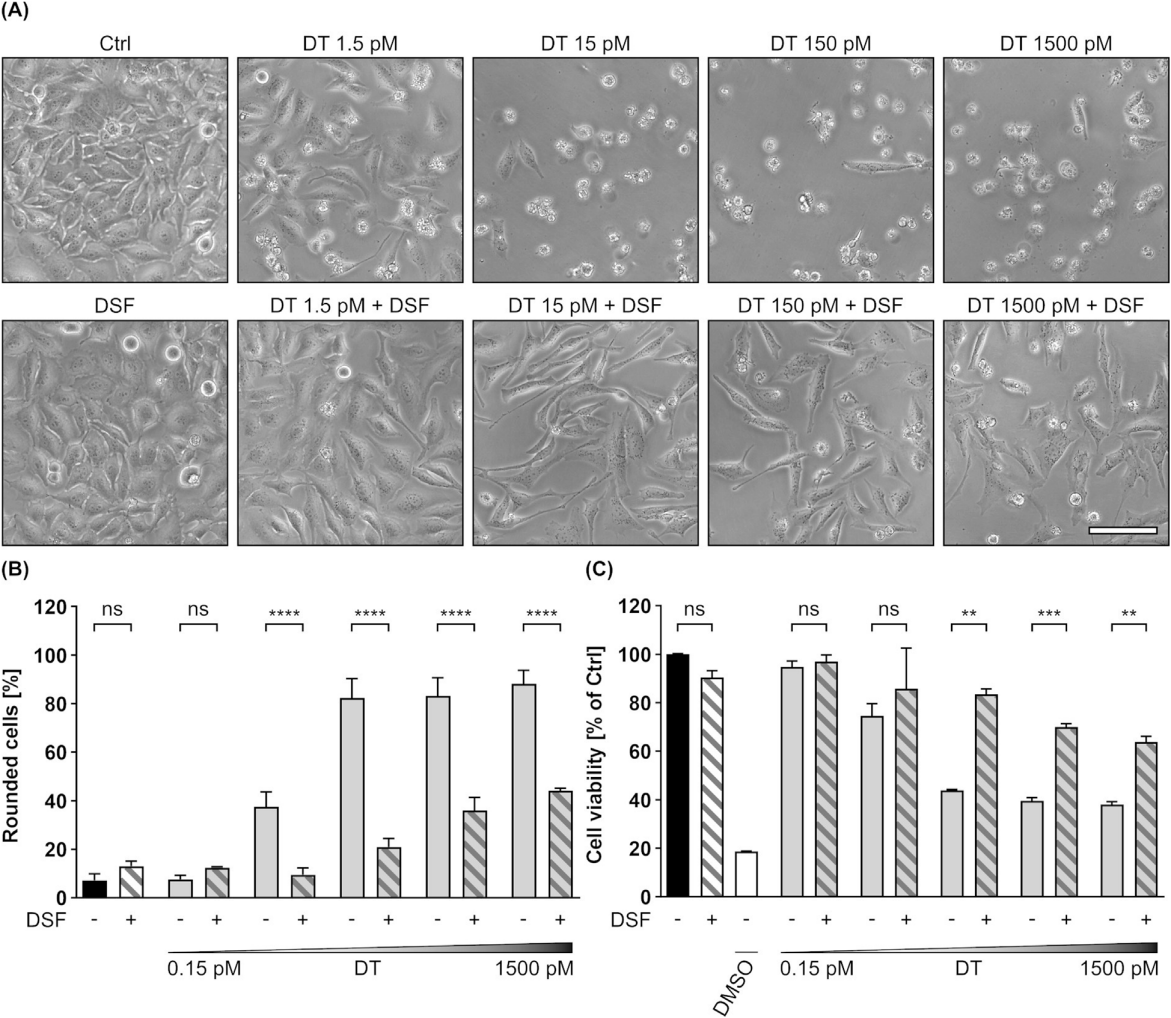
DSF prevents HeLa cells from toxic effects on their morphology and cell viability caused by DT. **(A)** Representative pictures after 24 h incubation time under the indicated conditions. DSF concentration was 15 µM. Sale bar corresponds to 100 µm. **(B)** Quantitative evaluation of cell rounding after 24 h incubation with 15 µM DSF and 0.15 p.m., 1.5 p.m., 15 p.m., 150 p.m. or 1500 p.m. DT in the indicated combinations, or without any compound as control (Ctrl). Values are given as mean ± SD (n = 3) of triplicates from one representative experiment of three independent replicates. Statistical analysis was performed as indicated by black brackets using one-way ANOVA with Šídák’s multiple comparison test (ns *p* ≥ 0.05, ****p < 0.0001). **(C)** Relative viability (% of Ctrl) of the cells from **(B)** measured via MTS assay. As an additional control for reduced viability, cells were incubated with toxic concentrations (20%) of DMSO. Values are given as mean ± SD (n = 3) of triplicates from one representative experiment of three independent replicates. Statistical analysis was performed as indicated by black brackets using one-way ANOVA with Šídák’s multiple comparison test (ns *p* ≥ 0.05, **p < 0.01, ***p < 0.001).

The results shown in this work so far imply, that DSF inhibits the intoxication of mammalian cells by the two related binary AB-type toxins C2 toxin and LT (or PA_83_/LF_N_-DTA, PA_83_/His-DTA). Moreover, this inhibition seems to be driven to a major extend by an interference of DSF with the membrane translocation of these toxins. Since membrane translocation is a central step in the intoxication process of many AB toxins, it was furthermore tested whether DSF also affects this step in a single-chain AB toxin. As it has already been shown that DSF does not influence the enzyme activity of DTA (see Figure 6B), DT was chosen as a model toxin for monomeric AB toxins. To elucidate potential inhibitory effects of DSF towards DT, HeLa cells were incubated for 24 h with increasing concentrations of DT in the presence or absence of DSF. Further cells were treated only with DSF or without any compound as control. As shown in Figures 8A–C, DT treatment led to a concentration-dependent increase in cell rounding and decrease in cell viability. However, both of these characteristic intoxication effects were significantly reduced in the presence of DSF. Even in the highest DT concentration used, DSF could reduce the portion of rounded cells by more than half and strongly lower the DT-mediated loss in cell viability. As it was also shown that the DSF solvent EtOH did not affect the intoxication of HeLa cells by DT (Supplementary Figures 2E, F), these results indicate that the observed inhibition of DT is driven by DSF.

### 3.7 DSF has no effect on DT binding to HeLa cells, but impairs its translocation

**FIGURE 9:**
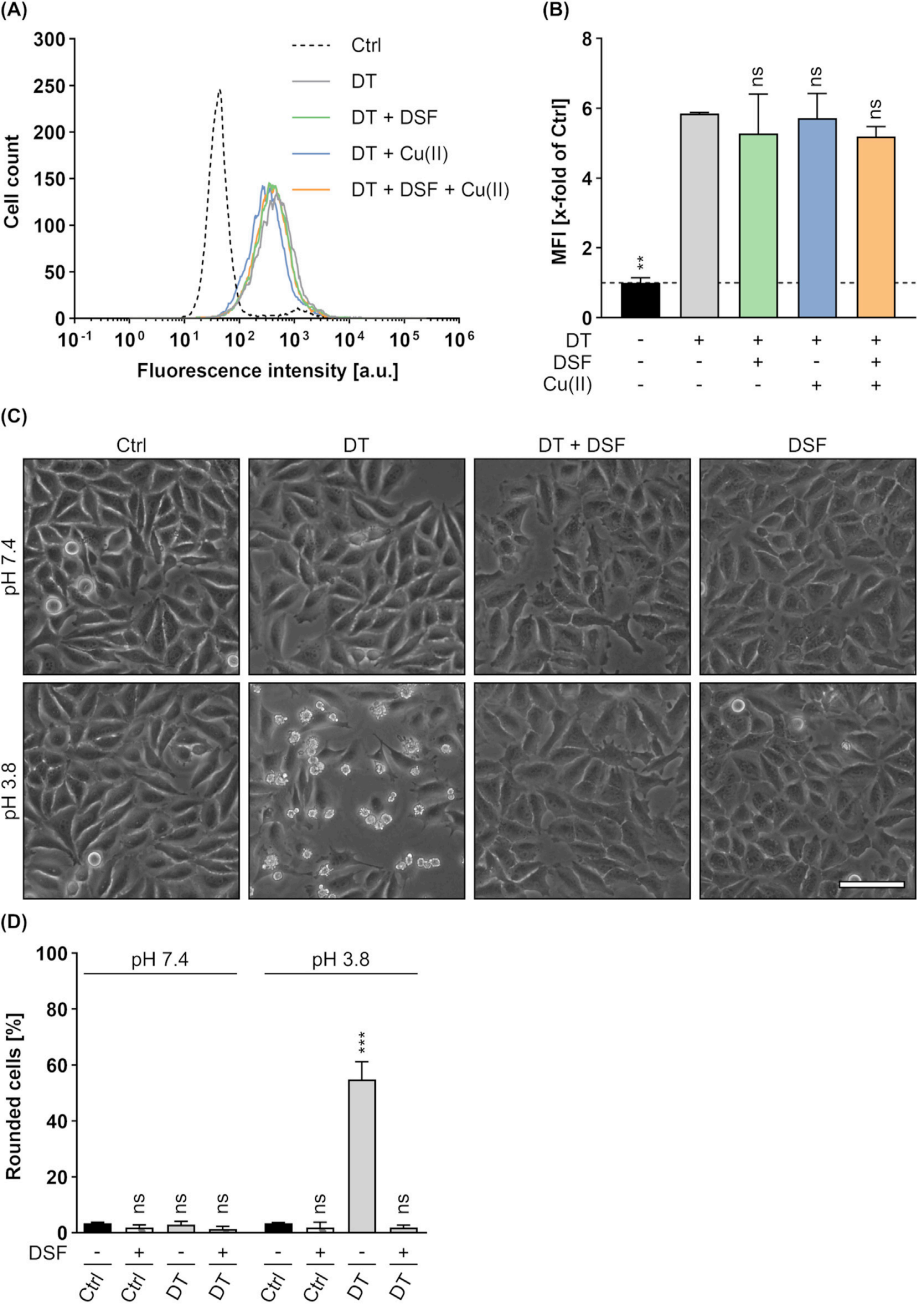
Effect of DSF on the cellular binding and translocation of DT. To analyze the binding of DT (250 nM) to HeLa cells in the presence or absence of 15 µM DSF and 330 nM Cu(II), flow cytometric analysis was performed and cell-bound DT was detected via specific antibodies. Control (Ctrl) cells were treated with PBS. **(A)** Representative histogram of fluorescence intensity values for the DT signal. **(B)** The MFI values of the DT signal of all samples were measured and normalized to the Ctrl. Values are given as mean ± SD (n = 2) of duplicates from one representative experiment of three independent replicates. Statistical analysis was performed and conditions were compared to the DT only condition by using one-way ANOVA with Dunnett’s correction for multiple comparison (ns *p* ≥ 0.05, **p < 0.01). **(C)** Assay for translocation across the plasma membrane via acidic pulse. HeLa cells were treated according to the assay protocol with 30 nM DT, 15 µM DSF, or without any compound as control (Ctrl). Pictures were taken 5 h after the acidic pulse and representative pictures are depicted. The upper panel shows cells pulsed with medium at pH 7.4, while the lower panel shows cells pulsed with medium at pH 3.8. Scale bar corresponds to 100 µm. **(D)** Quantification of the assay for translocation from **(C)**. Shown is the percentage of rounded cells 5 h after the acidic pulse. Data were obtained within the same experiment as the data from Figures 7A,B. Values are given as mean ± SD (n = 2) of duplicates from one representative experiment of three independent replicates. Statistical analysis was performed compared to the respective Ctrl (−) at pH 7.4 or pH 3.8 by using one-way ANOVA with Dunnett’s correction for multiple comparison (ns *p* ≥ 0.05, ***p < 0.001).

With respect to the observation that DSF inhibits the intoxication of cells by DT, the underlying mode of inhibition was investigated in more detail. Since it was already shown, that DSF does not influence the DTA activity (see Figure 6B), potential effects of DSF on cell-binding of DT were analyzed by flow cytometry. To this end, HeLa cells were incubated on ice (allows binding but not uptake) with DT in the presence or absence of DSF and/or Cu(II). Additional cells were treated with PBS and served as control for signal normalization (Figures 9A,B). After removal of unbound DT by washing, cell-bound DT was labelled with antibodies and analyzed by flow cytometry. Thereby, fluorescence intensities correlate with the amount of bound DT. As depicted in Figures 9A,B, neither DSF nor Cu(II) or their combination showed any effect on the binding of DT to HeLa cells. Hence, the inhibition of DT by DSF seems not to be based on an effect of DSF on DT binding. Finally, potential effects of DSF on the DT translocation were investigated by a translocation assay similar as described earlier (Section 3.2). While DT-treated cells showed a clear and expected rounding 5 h after acidification, this assay revealed a complete protection of the cells from DT-mediated rounding when DSF was present (Figures 9C,D). Cells treated with DT and DSF show a similar morphology as untreated cells and cells treated with DSF only. This result is comparable to the results obtained for C2 toxin (see Figures 3A,B) and PA_63_/LF_N_-DTA (see Figures 7A,B) in this assay and strongly indicates an inhibitory effect of DSF on the translocation process of DT, as it was also the case for the other investigated toxins in this study.

## 4 Discussion

Drug repurposing (drug repositioning) evaluates established, licensed pharmaceutical compounds for “off-target” effects to identify potential new therapeutic indications. This approach is in particular attractive as it is fast and comparatively cheap because the safety profile (toxicology) and the pharmacokinetic parameters for such compounds are already known. Therefore, this approach is particularly interesting for the development of therapeutic options against rare diseases where cost-intensive drug development might not be cost-effective, or for diseases where treatment options are urgently needed (Kulkarni et al., 2023). We and others exploited drug repositioning to identify pharmacological inhibitors against bacterial protein exotoxins, such as the medically highly relevant toxins from the pathogenic bacterium *Clostridioides* (*C.*) *difficile* (Tam et al., 2018; Zhu et al., 2019; Heber et al., 2022; Schumacher et al., 2023), DT (Sanchez et al., 2007; Schnell et al., 2016), pertussis toxin (Ernst et al., 2018), and binary *bacillus* and clostridial exotoxins (Sanchez et al., 2007; Slater et al., 2013; Kreidler et al., 2017; Ernst et al., 2021; Braune-Yan et al., 2023; Jia et al., 2023). Compounds that inhibit bacterial protein toxins usually either prevent the binding of the toxins to their target cell receptors, the transport of their A-subunits from endosomal vesicles into the host cell cytosol, or the enzyme activity of the A-subunits. Consequently, cells are protected from intoxication and toxin-associated diseases can be avoided. The use of different agents and especially licensed small molecular drugs that directly target protein toxins as the primary virulence factors of bacterial pathogens as an alternative or addition to existing treatment strategies is extensively investigated and the advantages and drawbacks of this approach have been excellently reviewed (Garland et al., 2017; Schein, 2020; Sakari et al., 2022).

In the present study, we found that DSF protects cells from DT, C2 toxin and anthrax lethal toxin when applied in concentrations lower than the maximal plasma concentration (20 µM) of adults receiving the typical DSF dose of 500 mg/day to treat alcoholism (Beaudry et al., 2023). This finding is supported by an earlier report that identified DSF as a potential inhibitor of LT in a small molecule screen (Slater et al., 2013). However, no inhibition of DSF towards C2 toxin and DT was tested so far and the mechanism by which DSF inhibits bacterial toxins was not investigated further. Here, a set of experiments was performed to study the molecular mechanism underlying the protective effect of DSF towards each of these toxins. DSF had no relevant effect on toxin binding to the cells and enzyme activity of the toxins, but prevented cells from intoxication, when the B-subunit-dependent, cytosolic translocation of the toxins’ A-subunits across the cell membrane was triggered by low pH. Moreover*, in vitro*, DSF blocked the conductance of pores formed by the purified toxin B-subunits in lipid membranes. Taken all together, our results implicate that DSF interferes with the trans-membrane-pores of the toxins, thereby preventing membrane translocation of the respective A-subunits into the cytosol of their target cells. Although the single-chain DT and the binary C2 and anthrax toxins differ in their structures, their A-subunits and their cellular substrate molecules, they share a widely common cellular uptake mechanism, which includes a central intracellular transport process of the respective A-subunits through a trans-membrane pore formed by specific subunits of the toxins across the membranes of acidified endosomal vesicles (Chenal et al., 2002; Blöcker et al., 2003a; Hardenbrook et al., 2020). The pores formed by DT, C2IIa and PA_63_ under acidic conditions therefore represent central drug targets. Since the process of toxin translocation represents a bottle neck during the cellular uptake of various bacterial toxins and its inhibition protects cells from the respective toxins (Bronnhuber et al., 2014; Kreidler et al., 2017), DSF might act as a pan-toxin-inhibitor applicable against a broad spectrum of bacterial protein toxins.

Following its re-discovery in the 1940s and approval by the FDA, DSF was used for more than 60 years for the treatment of chronic alcohol-abuse (Wright and Moore, 1990; Suh et al., 2006; Burnette et al., 2022). In this context, a metabolite of DSF covalently binds to and inhibits aldehyde-dehydrogenase (ALDH), which leads to accumulation of the toxic metabolite acetaldehyde after alcohol consumption, resulting in unpleasant physiological effects (Vallari and Pietruszko, 1982; Burnette et al., 2022). However, side-effects were observed that correlated with the proportions of consumed alcohol and the DSF dose (Suh et al., 2006), and because there is inter-individual variability in DSF pharmacokinetics (Lanz et al., 2023), concern raised regarding the safety profile of DSF after prolonged treatment. Therefore, DSF is no longer used in many countries for its original purpose but there is increasing evidence from drug-repurposing studies that DSF might be interesting for novel indications apart from alcohol-abuse. Potential applications of DSF besides the here presented inhibition of bacterial protein toxins are infections with viruses, parasites or bacteria (e.g., *Staphylococcus aureus*, *Borrelia burgdorferi*) (Lanz et al., 2023), inflammation (Hu et al., 2020), cancer (e.g., neuroblastoma) (Skrott et al., 2017; Beaudry et al., 2023), neurological diseases, and addictions (e.g., cocaine) (Lanz et al., 2023). Thus, despite the risk concerns which mainly are associated with alcohol metabolism (Suh et al., 2006), DSF might be an attractive option for the treatment of various diseases, in particular when specific risk factors such as alcohol consumption can be avoided during DSF treatment. Moreover, since we demonstrated that Cu(II) enhances the inhibitory activity of DSF towards C2 toxin and LT and allows reduction of the DSF concentration while keeping a high level of inhibition, the risk of dose-correlated side-effects of DSF might be further minimized by reduction of DSF doses in combination with Cu(II). This is especially interesting since several clinical trials already demonstrated safety and tolerability for combinations of DSF and Cu(II) in patients (Huang et al., 2019; Kelley et al., 2021).

## Data Availability

The original contributions presented in the study are included in the article/Supplementary Material, further inquiries can be directed to the corresponding author.

## References

[B1] Aktories K. Bärmann M. Ohishi I. Tsuyama S. Jakobs K. H. Habermann E. (1986). Botulinum C2 toxin ADP-ribosylates actin. Nature 322, 390–392. 10.1038/322390a0 3736664

[B2] Aktories K. Reuner K.-H. Presek P. Bärmann M. (1989). Botulinum C2 toxin treatment increases the G-actin pool in intact chicken cells: a model for the cytopathic action of actin-ADP-ribosylation toxins. Toxicon 27, 989–993. 10.1016/0041-0101(89)90149-9 2508273

[B3] Arora N. Leppla S. H. (1994). Fusions of anthrax toxin lethal factor with shiva toxin and diphtheria toxin enzymatic domains are toxic to mammalian cells. Infect. Immun. 62, 4955–4961. 10.1128/iai.62.11.4955-4961.1994 7927776 PMC303212

[B4] Baaken G. Sondermann M. Schlemmer C. Rühe J. Behrends J. C. (2008). Planar microelectrode-cavity array for high-resolution and parallel electrical recording of membrane ionic currents. Lab. Chip 8, 938–944. 10.1039/b800431e 18497915

[B5] Bachmeyer C. Benz R. Barth H. Aktories K. Gibert M. R. Popoff M. (2001). Interaction of *Clostridium botulinum* C2‐toxin with lipid bilayer membranes and vero cells: inhibition of channel function by chloroquine and related compounds *in vitro* and intoxication *in vivo* . FASEB J. 15, 1658–1660. 10.1096/fj.00-0671fje 11427518

[B6] Bargis‐Surgey P. Lavergne J. Gonzalo P. Vard C. Filhol‐Cochet O. Reboud J. (1999). Interaction of elongation factor eEF‐2 with ribosomal P proteins. Eur. J. Biochem. 262, 606–611. 10.1046/j.1432-1327.1999.00434.x 10336649

[B7] Barth H. Aktories K. Popoff M. R. Stiles B. G. (2004). Binary bacterial toxins: biochemistry, biology, and applications of common *Clostridium* and *Bacillus* proteins. Microbiol. Mol. Biol. Rev. 68, 373–402. 10.1128/MMBR.68.3.373-402.2004 15353562 PMC515256

[B8] Barth H. Blöcker D. Behlke J. Bergsma-Schutter W. Brisson A. Benz R. (2000). Cellular uptake of Clostridium botulinum C2 toxin requires oligomerization and acidification. J. Biol. Chem. 275, 18704–18711. 10.1074/jbc.M000596200 10749859

[B9] Barth H. Hofmann F. Olenik C. Just I. Aktories K. (1998a). The N-terminal part of the enzyme component (C2I) of the binary *Clostridium botulinum* C2 toxin interacts with the binding component C2II and functions as a carrier system for a rho ADP-ribosylating C3-like fusion toxin. Infect. Immun. 66, 1364–1369. 10.1128/IAI.66.4.1364-1369.1998 9529054 PMC108061

[B10] Barth H. Preiss J. C. Hofmann F. Aktories K. (1998b). Characterization of the catalytic site of the ADP-ribosyltransferase Clostridium botulinum C2 toxin by site-directed mutagenesis. J. Biol. Chem. 273, 29506–29511. 10.1074/jbc.273.45.29506 9792657

[B11] Beaudry A. Jacques-Ricard S. Darracq A. Sgarioto N. Garcia A. García T. R. (2023). Repurposing disulfiram, an alcohol-abuse drug, in neuroblastoma causes KAT2A downregulation and *in vivo* activity with a water/oil emulsion. Sci. Rep. 13, 16443. 10.1038/s41598-023-43219-2 37777587 PMC10543387

[B12] Bhatnagar R. Ahuja N. Goila R. Batra S. Waheed S. M. Gupta P. (1999). Activation of phospholipase C and protein kinase C is required for expression of anthrax lethal toxin cytotoxicity in J774A.1 cells. Cell. Signal. 11, 111–116. 10.1016/S0898-6568(98)00041-2 10048788

[B13] Blanke S. R. Milne J. C. Benson E. L. Collier R. J. (1996). Fused polycationic peptide mediates delivery of diphtheria toxin A chain to the cytosol in the presence of anthrax protective antigen. Proc. Natl. Acad. Sci. U.S.A. 93, 8437–8442. 10.1073/pnas.93.16.8437 8710889 PMC38689

[B14] Blaustein R. O. Koehler T. M. Collier R. J. Finkelstein A. (1989). Anthrax toxin: channel-forming activity of protective antigen in planar phospholipid bilayers. Proc. Natl. Acad. Sci. U.S.A. 86, 2209–2213. 10.1073/pnas.86.7.2209 2467303 PMC286881

[B15] Blewitt M. G. Chung L. A. London E. (1985). Effect of pH on the conformation of diphtheria toxin and its implications for membrane penetration. Biochemistry 24, 5458–5464. 10.1021/bi00341a027 4074708

[B16] Blöcker D. Bachmeyer C. Benz R. Aktories K. Barth H. (2003a). Channel formation by the binding component of *Clostridium botulinum* C2 toxin: glutamate 307 of C2II affects Channel properties *in vitro* and pH-dependent C2I translocation *in vivo* . Biochemistry 42, 5368–5377. 10.1021/bi034199e 12731878

[B17] Blöcker D. Barth H. Maier E. Benz R. Barbieri J. T. Aktories K. (2000). The C terminus of component C2II of *Clostridium botulinum* C2 toxin is essential for receptor binding. Infect. Immun. 68, 4566–4573. 10.1128/IAI.68.8.4566-4573.2000 10899856 PMC98375

[B18] Blöcker D. Pohlmann K. Haug G. Bachmeyer C. Benz R. Aktories K. (2003b). Clostridium botulinum C2 toxin: low pH-induced pore formation is required for translocation of the enzyme component C2I into the cytosol of host cells. J. Biol. Chem. 278, 37360–37367. 10.1074/jbc.M305849200 12869543

[B19] Bradley K. A. Mogridge J. Mourez M. Collier R. J. Young J. A. T. (2001). Identification of the cellular receptor for anthrax toxin. Nature 414, 225–229. 10.1038/n35101999 11700562

[B20] Braune-Yan M. Jia J. Wahba M. Schmid J. Papatheodorou P. Barth H. (2023). Domperidone protects cells from intoxication with Clostridioides difficile toxins by inhibiting hsp70-assisted membrane translocation. Toxins 15, 384. 10.3390/toxins15060384 37368685 PMC10305128

[B21] Bronnhuber A. Maier E. Riedl Z. Hajós G. Benz R. Barth H. (2014). Inhibitions of the translocation pore of Clostridium botulinum C2 toxin by tailored azolopyridinium salts protects human cells from intoxication. Toxicology 316, 25–33. 10.1016/j.tox.2013.12.006 24394545

[B22] Burnette E. M. Nieto S. J. Grodin E. N. Meredith L. R. Hurley B. Miotto K. (2022). Novel agents for the pharmacological treatment of alcohol use disorder. Drugs 82, 251–274. 10.1007/s40265-021-01670-3 35133639 PMC8888464

[B23] Castillo-Villanueva A. Rufino-González Y. Méndez S.-T. Torres-Arroyo A. Ponce-Macotela M. Martínez-Gordillo M. N. (2017). Disulfiram as a novel inactivator of Giardia lamblia triosephosphate isomerase with antigiardial potential. Int. J. Parasitol. Drugs Drug Resist. 7, 425–432. 10.1016/j.ijpddr.2017.11.003 29197728 PMC5727346

[B24] Chenal A. Nizard P. Gillet D. (2002). Structure and function of diphtheria toxin: from pathology to engineering. J. Toxicol. Toxin Rev. 21, 321–359. 10.1081/TXR-120014408

[B25] Choe S. Bennett M. J. Fujii G. Curmi P. M. G. Kantardjieff K. A. Collier R. J. (1992). The crystal structure of diphtheria toxin. Nature 357, 216–222. 10.1038/357216a0 1589020

[B26] Collier R. J. (1967). Effect of diphtheria toxin on protein synthesis: inactivation of one of the transfer factors. J. Mol. Biol. 25, 83–98. 10.1016/0022-2836(67)90280-X 4291872

[B27] Collier R. J. (2001). Understanding the mode of action of diphtheria toxin: a perspective on progress during the 20th century. Toxicon 39, 1793–1803. 10.1016/S0041-0101(01)00165-9 11595641

[B28] Collier R. J. (2009). Membrane translocation by anthrax toxin. Mol. Aspects Med. 30, 413–422. 10.1016/j.mam.2009.06.003 19563824 PMC2783560

[B29] Collier R. J. Cole H. A. (1969). Diphtheria toxin submit active *in vitro* . Science 164, 1179–1182. 10.1126/science.164.3884.1179 4305968

[B30] Donovan J. J. Simon M. I. Draper R. K. Montal M. (1981). Diphtheria toxin forms transmembrane channels in planar lipid bilayers. Proc. Natl. Acad. Sci. U.S.A. 78, 172–176. 10.1073/pnas.78.1.172 6264431 PMC319013

[B31] Eckhardt M. Barth H. Blöcker D. Aktories K. (2000). Binding of Clostridium botulinum C2 toxin to asparagine-linked complex and hybrid carbohydrates. J. Biol. Chem. 275, 2328–2334. 10.1074/jbc.275.4.2328 10644682

[B32] Eisele J. Schreiner S. Borho J. Fischer S. Heber S. Endres S. (2022). The pore-forming subunit C2IIa of the binary Clostridium botulinum C2 toxin reduces the chemotactic translocation of human polymorphonuclear leukocytes. Front. Pharmacol. 13, 810611. 10.3389/fphar.2022.810611 35222028 PMC8881014

[B33] Ernst K. Eberhardt N. Mittler A.-K. Sonnabend M. Anastasia A. Freisinger S. (2018). Pharmacological cyclophilin inhibitors prevent intoxication of mammalian cells with bordetella pertussis toxin. Toxins 10, 181. 10.3390/toxins10050181 29723951 PMC5983237

[B34] Ernst K. Landenberger M. Nieland J. Nørgaard K. Frick M. Fois G. (2021). Characterization and pharmacological inhibition of the pore-forming Clostridioides difficile CDTb toxin. Toxins 13, 390. 10.3390/toxins13060390 34071730 PMC8226936

[B35] Falnes P. Ø. Sandvig K. (2000). Penetration of protein toxins into cells. Curr. Opin. Cell. Biol. 12, 407–413. 10.1016/S0955-0674(00)00109-5 10873820

[B36] Gajdács M. Albericio F. (2019). Antibiotic resistance: from the bench to patients. Antibiotics 8, 129. 10.3390/antibiotics8030129 31461842 PMC6783868

[B37] Garland M. Loscher S. Bogyo M. (2017). Chemical strategies to target bacterial virulence. Chem. Rev. 117, 4422–4461. 10.1021/acs.chemrev.6b00676 28234447

[B38] Greenfield L. Bjorn M. J. Horn G. Fong D. Buck G. A. Collier R. J. (1983). Nucleotide sequence of the structural gene for diphtheria toxin carried by corynebacteriophage beta. Proc. Natl. Acad. Sci. U.S.A. 80, 6853–6857. 10.1073/pnas.80.22.6853 6316330 PMC390084

[B39] Hardenbrook N. J. Liu S. Zhou K. Ghosal K. Zhou Z. H. Krantz B. A. (2020). Atomic structures of anthrax toxin protective antigen channels bound to partially unfolded lethal and edema factors. Nat. Commun. 11, 840. 10.1038/s41467-020-14658-6 32047164 PMC7012834

[B40] Heber S. Barthold L. Baier J. Papatheodorou P. Fois G. Frick M. (2022). Inhibition of Clostridioides difficile toxins TcdA and TcdB by ambroxol. Front. Pharmacol. 12, 809595. 10.3389/fphar.2021.809595 35058787 PMC8764291

[B41] Heber S. Borho J. Stadler N. Wondany F. König I. Michaelis J. (2023). The Clostridium botulinum C2 toxin subunit C2IIa delivers enzymes with positively charged N-termini into the cytosol of target cells. Toxins 15, 390. 10.3390/toxins15060390 37368691 PMC10305195

[B42] Heine K. Pust S. Enzenmüller S. Barth H. (2008). ADP-ribosylation of actin by the *Clostridium botulinum* C2 toxin in mammalian cells results in delayed caspase-dependent apoptotic cell death. Infect. Immun. 76, 4600–4608. 10.1128/IAI.00651-08 18710868 PMC2546856

[B43] Holmes R. K. (2000). Biology and molecular epidemiology of diphtheria toxin and the *tox* gene. J. Infect. Dis. 181, S156–S167. 10.1086/315554 10657208

[B44] Honjo T. Nishizuka Y. Hayaishi O. Kato I. (1968). Diphtheria toxin-dependent adenosine diphosphate ribosylation of aminoacyl transferase II and inhibition of protein synthesis. J. Biol. Chem. 243, 3553–3555. 10.1016/s0021-9258(18)93347-8 4297784

[B45] Hu J. J. Liu X. Xia S. Zhang Z. Zhang Y. Zhao J. (2020). FDA-approved disulfiram inhibits pyroptosis by blocking gasdermin D pore formation. Nat. Immunol. 21, 736–745. 10.1038/s41590-020-0669-6 32367036 PMC7316630

[B46] Huang J. Chaudhary R. Cohen A. L. Fink K. Goldlust S. Boockvar J. (2019). A multicenter phase II study of temozolomide plus disulfiram and copper for recurrent temozolomide-resistant glioblastoma. J. Neurooncol 142, 537–544. 10.1007/s11060-019-03125-y 30771200

[B47] Iwamoto R. Higashiyama S. Mitamura T. Taniguchi N. Klagsbrun M. Mekada E. (1994). Heparin-binding EGF-like growth factor, which acts as the diphtheria toxin receptor, forms a complex with membrane protein DRAP27/CD9, which up-regulates functional receptors and diphtheria toxin sensitivity. EMBO J. 13, 2322–2330. 10.1002/j.1460-2075.1994.tb06516.x 8194524 PMC395097

[B48] Jia J. Braune-Yan M. Lietz S. Wahba M. Pulliainen A. T. Barth H. (2023). Domperidone inhibits Clostridium botulinum C2 toxin and bordetella pertussis toxin. Toxins 15, 412. 10.3390/toxins15070412 37505681 PMC10467066

[B49] Jiang J. Pentelute B. L. Collier R. J. Zhou Z. H. (2015). Atomic structure of anthrax protective antigen pore elucidates toxin translocation. Nature 521, 545–549. 10.1038/nature14247 25778700 PMC4519040

[B50] Kagan B. L. Finkelstein A. Colombini M. (1981). Diphtheria toxin fragment forms large pores in phospholipid bilayer membranes. Proc. Natl. Acad. Sci. U.S.A. 78, 4950–4954. 10.1073/pnas.78.8.4950 6272284 PMC320306

[B51] Kaiser E. Haug G. Hliscs M. Aktories K. Barth H. (2006). Formation of a biologically active toxin complex of the binary *Clostridium botulinum* C2 toxin without cell membrane interaction. Biochemistry 45, 13361–13368. 10.1021/bi061459u 17073457

[B52] Kang X. Jadhav S. Annaji M. Huang C.-H. Amin R. Shen J. (2023). Advancing cancer therapy with copper/disulfiram nanomedicines and drug delivery systems. Pharmaceutics 15, 1567. 10.3390/pharmaceutics15061567 37376016 PMC10302862

[B53] Keen J. H. Maxfield F. R. Hardegree M. C. Habig W. H. (1982). Receptor-mediated endocytosis of diphtheria toxin by cells in culture. Proc. Natl. Acad. Sci. U.S.A. 79, 2912–2916. 10.1073/pnas.79.9.2912 6178112 PMC346318

[B54] Kelley K. C. Grossman K. F. Brittain-Blankenship M. Thorne K. M. Akerley W. L. Terrazas M. C. (2021). A Phase 1 dose-escalation study of disulfiram and copper gluconate in patients with advanced solid tumors involving the liver using S-glutathionylation as a biomarker. BMC Cancer 21, 510. 10.1186/s12885-021-08242-4 33957901 PMC8103752

[B55] Kling C. Sommer A. Almeida-Hernandez Y. Rodríguez A. Perez-Erviti J. A. Bhadane R. (2023). Inhibition of pertussis toxin by human α-defensins-1 and -5: differential mechanisms of action. IJMS 24, 10557. 10.3390/ijms241310557 37445740 PMC10341622

[B56] Knapp O. Benz R. Popoff M. R. (2016). Pore-forming activity of clostridial binary toxins. Biochimica Biophysica Acta (BBA) - Biomembr. 1858, 512–525. 10.1016/j.bbamem.2015.08.006 26278641

[B57] Krantz B. A. Melnyk R. A. Zhang S. Juris S. J. Lacy D. B. Wu Z. (2005). A phenylalanine clamp catalyzes protein translocation through the anthrax toxin pore. Science 309, 777–781. 10.1126/science.1113380 16051798 PMC1815389

[B58] Kreidler A.-M. Benz R. Barth H. (2017). Chloroquine derivatives block the translocation pores and inhibit cellular entry of Clostridium botulinum C2 toxin and Bacillus anthracis lethal toxin. Arch. Toxicol. 91, 1431–1445. 10.1007/s00204-016-1716-9 27106023

[B59] Kulkarni V. S. Alagarsamy V. Solomon V. R. Jose P. A. Murugesan S. (2023). Drug repurposing: an effective tool in modern drug discovery. Russ. J. Bioorg Chem. 49, 157–166. 10.1134/S1068162023020139 36852389 PMC9945820

[B60] Kurazono H. Hosokawa M. Matsuda H. Sakaguchi G. (1987). Fluid accumulation in the ligated intestinal loop and histopathological changes of the intestinal mucosa caused by Clostridium botulinum C2 toxin in the pheasant and chicken. Res. Vet. Sci. 42, 349–353. 10.1016/s0034-5288(18)30717-3 3616149

[B61] Lacy D. B. Wigelsworth D. J. Melnyk R. A. Harrison S. C. Collier R. J. (2004). Structure of heptameric protective antigen bound to an anthrax toxin receptor: a role for receptor in pH-dependent pore formation. Proc. Natl. Acad. Sci. U.S.A. 101, 13147–13151. 10.1073/pnas.0405405101 15326297 PMC516539

[B62] Lang A. E. Neumeyer T. Sun J. Collier R. J. Benz R. Aktories K. (2008). Amino acid residues involved in membrane insertion and pore formation of *Clostridium botulinum* C2 toxin. Biochemistry 47, 8406–8413. 10.1021/bi800615g 18636745

[B63] Lanz J. Biniaz-Harris N. Kuvaldina M. Jain S. Lewis K. Fallon B. A. (2023). Disulfiram: mechanisms, applications, and challenges. Antibiotics 12, 524. 10.3390/antibiotics12030524 36978391 PMC10044060

[B64] Laxminarayan R. Duse A. Wattal C. Zaidi A. K. M. Wertheim H. F. L. Sumpradit N. (2013). Antibiotic resistance—the need for global solutions. Lancet Infect. Dis. 13, 1057–1098. 10.1016/S1473-3099(13)70318-9 24252483

[B65] Lemichez E. Bomsel M. Devilliers G. Van Der Spek J. Murphy J. R. Lukianov E. V. (1997). Membrane translocation of diphtheria toxin fragment A exploits early to late endosome trafficking machinery. Mol. Microbiol. 23, 445–457. 10.1111/j.1365-2958.1997.tb02669.x 9044279

[B66] Lin C.-G. Kao Y.-T. Liu W.-T. Huang H.-H. Chen K.-C. Wang T.-M. (1996). Cytotoxic effects of anthrax lethal toxin on macrophage-like cell line J774A.1. Curr. Microbiol. 33, 224–227. 10.1007/s002849900104 8824167

[B67] Lord J. M. Smith D. C. Roberts L. M. (1999). Toxin entry: how bacterial proteins get into mammalian cells. Cell. Microbiol. 1, 85–91. 10.1046/j.1462-5822.1999.00015.x 11207543

[B68] Lu C. Li X. Ren Y. Zhang X. (2021). Disulfiram: a novel repurposed drug for cancer therapy. Cancer Chemother. Pharmacol. 87, 159–172. 10.1007/s00280-020-04216-8 33426580

[B69] Melnyk R. A. Hewitt K. M. Lacy D. B. Lin H. C. Gessner C. R. Li S. (2006). Structural determinants for the binding of anthrax lethal factor to oligomeric protective antigen. J. Biol. Chem. 281, 1630–1635. 10.1074/jbc.M511164200 16293620

[B70] Milne J. C. Collier R. J. (1993). pH‐dependent permeabilization of the plasma membrane of mammalian cells by anthrax protective antigen. Mol. Microbiol. 10, 647–653. 10.1111/j.1365-2958.1993.tb00936.x 7968541

[B71] Mitamura T. Higashiyama S. Taniguchi N. Klagsbrun M. Mekada E. (1995). Diphtheria toxin binds to the epidermal growth factor (EGF)-like domain of human heparin-binding EGF-like growth factor/diphtheria toxin receptor and inhibits specifically its mitogenic activity. J. Biol. Chem. 270, 1015–1019. 10.1074/jbc.270.3.1015 7836353

[B72] Moayeri M. Leppla S. H. Vrentas C. Pomerantsev A. P. Liu S. (2015). Anthrax pathogenesis. Annu. Rev. Microbiol. 69, 185–208. 10.1146/annurev-micro-091014-104523 26195305

[B73] Montecucco C. (1998). Protein toxins and membrane transport. Curr. Opin. Cell. Biol. 10, 530–536. 10.1016/S0955-0674(98)80069-0 9719875

[B74] Murphy J. R. (2011). Mechanism of diphtheria toxin catalytic domain delivery to the eukaryotic cell cytosol and the cellular factors that directly participate in the process. Toxins 3, 294–308. 10.3390/toxins3030294 22069710 PMC3202816

[B75] Nagahama M. Hagiyama T. Kojima T. Aoyanagi K. Takahashi C. Oda M. (2009). Binding and internalization of *Clostridium botulinum* C2 toxin. Infect. Immun. 77, 5139–5148. 10.1128/IAI.00638-09 19720757 PMC2772504

[B76] Naglich J. G. Metherall J. E. Russell D. W. Eidels L. (1992). Expression cloning of a diphtheria toxin receptor: identity with a heparin-binding EGF-like growth factor precursor. Cell. 69, 1051–1061. 10.1016/0092-8674(92)90623-K 1606612

[B77] Neumeyer T. Schiffler B. Maier E. Lang A. E. Aktories K. Benz R. (2008). Clostridium botulinum C2 Toxin. Identification of the binding site for chloroquine and related compounds and influence of the binding site on properties of the C2II channel. J. Biol. Chem. 283, 3904–3914. 10.1074/jbc.M709807200 18077455

[B78] Nobel C. S. I. Kimland M. Nicholson D. W. Orrenius S. Slater A. F. G. (1997). Disulfiram is a potent inhibitor of proteases of the caspase family. Chem. Res. Toxicol. 10, 1319–1324. 10.1021/tx970131m 9437520

[B79] Oh K. J. Senzel L. Collier R. J. Finkelstein A. (1999). Translocation of the catalytic domain of diphtheria toxin across planar phospholipid bilayers by its own T domain. Proc. Natl. Acad. Sci. U.S.A. 96, 8467–8470. 10.1073/pnas.96.15.8467 10411898 PMC17539

[B80] Ohishi I. (1983a). Lethal and vascular permeability activities of botulinum C2 toxin induced by separate injections of the two toxin components. Infect. Immun. 40, 336–339. 10.1128/iai.40.1.336-339.1983 6832833 PMC264853

[B81] Ohishi I. (1983b). Response of mouse intestinal loop to botulinum C2 toxin: enterotoxin activity induced by cooperation of nonlinked protein components. Infect. Immun. 40, 691–695. 10.1128/iai.40.2.691-695.1983 6341246 PMC264910

[B82] Ohishi I. (1987). Activation of botulinum C2 toxin by trypsin. Infect. Immun. 55, 1461–1465. 10.1128/iai.55.6.1461-1465.1987 3570475 PMC260537

[B83] Ohishi I. Miyake M. (1985). Binding of the two components of C2 toxin to epithelial cells and brush borders of mouse intestine. Infect. Immun. 48, 769–775. 10.1128/iai.48.3.769-775.1985 3888843 PMC261258

[B84] Ohishi I. Miyake M. Ogura H. Nakamura S. (1984). Cytopathic effect of botulinum C _2_ toxin on tissue-culture cells. FEMS Microbiol. Lett. 23, 281–284. 10.1111/j.1574-6968.1984.tb01078.x

[B85] Ohishi I. Yanagimoto A. (1992). Visualizations of binding and internalization of two nonlinked protein components of botulinum C2 toxin in tissue culture cells. Infect. Immun. 60, 4648–4655. 10.1128/iai.60.11.4648-4655.1992 1398978 PMC258214

[B86] Orlik F. Schiffler B. Benz R. (2005). Anthrax toxin protective antigen: inhibition of channel function by chloroquine and related compounds and study of binding kinetics using the current noise analysis. Biophysical J. 88, 1715–1724. 10.1529/biophysj.104.050336 PMC1305228 15596516

[B87] Papatheodorou P. Aktories K. (2016). “Receptor-binding and uptake of binary actin-ADP-ribosylating toxins,” in Uptake and trafficking of protein toxins. Editor Barth H. (Cham: Springer International Publishing), 119–133. 10.1007/82_2016_46 27817176

[B88] Papini E. Rappuoli R. Murgia M. Montecucco C. (1993). Cell penetration of diphtheria toxin. Reduction of the interchain disulfide bridge is the rate-limiting step of translocation in the cytosol. J. Biol. Chem. 268, 1567–1574. 10.1016/s0021-9258(18)53890-4 8420931

[B89] Papini E. Sandoná D. Rappuoli R. Montecucco C. (1988). On the membrane translocation of diphtheria toxin: at low pH the toxin induces ion channels on cells. EMBO J. 7, 3353–3359. 10.1002/j.1460-2075.1988.tb03207.x 2463157 PMC454832

[B90] Pappenheimer A. M. (1977). Diphtheria toxin. Annu. Rev. Biochem. 46, 69–94. 10.1146/annurev.bi.46.070177.000441 20040

[B91] Petosa C. Collier R. J. Klimpel K. R. Leppla S. H. Liddington R. C. (1997). Crystal structure of the anthrax toxin protective antigen. Nature 385, 833–838. 10.1038/385833a0 9039918

[B92] Popoff M. R. (2024). Overview of bacterial protein toxins from pathogenic bacteria: mode of action and insights into evolution. Toxins 16, 182. 10.3390/toxins16040182 38668607 PMC11054074

[B93] Pust S. Barth H. Sandvig K. (2010). Clostridium botulinum C2 toxin is internalized by clathrin- and Rho-dependent mechanisms: endocytic mechanisms underlying C2 toxin uptake. Cell. Microbiol. 12, 1809–1820. 10.1111/j.1462-5822.2010.01512.x 20690924

[B94] Rabideau A. E. Pentelute B. L. (2016). Delivery of non-native cargo into mammalian cells using anthrax lethal toxin. ACS Chem. Biol. 11, 1490–1501. 10.1021/acschembio.6b00169 27055654

[B95] Sakari M. Laisi A. Pulliainen A. T. (2022). Exotoxin-targeted drug modalities as antibiotic alternatives. ACS Infect. Dis. 8, 433–456. 10.1021/acsinfecdis.1c00296 35099182 PMC8922280

[B96] Sanchez A. M. Thomas D. Gillespie E. J. Damoiseaux R. Rogers J. Saxe J. P. (2007). Amiodarone and bepridil inhibit anthrax toxin entry into host cells. Antimicrob. Agents Chemother. 51, 2403–2411. 10.1128/AAC.01184-06 17485504 PMC1913235

[B97] Sandvig K. Olsnes S. (1980). Diphtheria toxin entry into cells is facilitated by low pH. J. Cell. Biol. 87, 828–832. 10.1083/jcb.87.3.828 7462324 PMC2110776

[B98] Sandvig K. Olsnes S. (1988). Diphtheria toxin-induced channels in Vero cells selective for monovalent cations. J. Biol. Chem. 263, 12352–12359. 10.1016/s0021-9258(18)37762-7 2457582

[B99] Schein C. H. (2020). Repurposing approved drugs on the pathway to novel therapies. Med. Res. Rev. 40, 586–605. 10.1002/med.21627 31432544 PMC7018532

[B100] Schleberger C. Hochmann H. Barth H. Aktories K. Schulz G. E. (2006). Structure and action of the binary C2 toxin from Clostridium botulinum. J. Mol. Biol. 364, 705–715. 10.1016/j.jmb.2006.09.002 17027031

[B101] Schmid A. Benz R. Just I. Aktories K. (1994). Interaction of Clostridium botulinum C2 toxin with lipid bilayer membranes. Formation of cation-selective channels and inhibition of channel function by chloroquine. J. Biol. Chem. 269, 16706–16711. 10.1016/s0021-9258(19)89448-6 7515883

[B102] Schmitt C. K. Meysick K. C. O’Brien A. D. (1999). Bacterial toxins: friends or foes? Emerg. Infect. Dis. 5, 224–234. 10.3201/eid0502.990206 10221874 PMC2640701

[B103] Schnell L. Dmochewitz-Kück L. Feigl P. Montecucco C. Barth H. (2016). Thioredoxin reductase inhibitor auranofin prevents membrane transport of diphtheria toxin into the cytosol and protects human cells from intoxication. Toxicon 116, 23–28. 10.1016/j.toxicon.2015.04.012 25911959

[B104] Schumacher J. Nienhaus A. Heber S. Matylitsky J. Chaves-Olarte E. Rodríguez C. (2023). Exploring the inhibitory potential of the antiarrhythmic drug amiodarone against *Clostridioides difficile* toxins TcdA and TcdB. Gut Microbes 15, 2256695. 10.1080/19490976.2023.2256695 37749884 PMC10524773

[B105] Scobie H. M. Rainey G. J. A. Bradley K. A. Young J. A. T. (2003). Human capillary morphogenesis protein 2 functions as an anthrax toxin receptor. Proc. Natl. Acad. Sci. U.S.A. 100, 5170–5174. 10.1073/pnas.0431098100 12700348 PMC154317

[B106] Singh Y. Chaudhary V. K. Leppla S. H. (1989). A deleted variant of Bacillus anthracis protective antigen is non-toxic and blocks anthrax toxin action *in vivo* . J. Biol. Chem. 264, 19103–19107. 10.1016/s0021-9258(19)47273-6 2509473

[B107] Skrott Z. Mistrik M. Andersen K. K. Friis S. Majera D. Gursky J. (2017). Alcohol-abuse drug disulfiram targets cancer via p97 segregate adaptor NPL4. Nature 552, 194–199. 10.1038/nature25016 29211715 PMC5730499

[B108] Slater L. H. Hett E. C. Mark K. Chumbler N. M. Patel D. Lacy D. B. (2013). Identification of novel host-targeted compounds that protect from anthrax lethal toxin-induced cell death. ACS Chem. Biol. 8, 812–822. 10.1021/cb300555n 23343607 PMC3638717

[B109] Sterthoff C. Lang A. E. Schwan C. Tauch A. Aktories K. (2010). Functional characterization of an extended binding component of the actin-ADP-ribosylating C2 toxin detected in *Clostridium botulinum* strain (C) 2300. Infect. Immun. 78, 1468–1474. 10.1128/IAI.01351-09 20145093 PMC2849413

[B110] Stiles B. Pradhan K. Fleming J. Samy R. Barth H. Popoff M. (2014). Clostridium and Bacillus binary enterotoxins: bad for the bowels, and eukaryotic being. Toxins 6, 2626–2656. 10.3390/toxins6092626 25198129 PMC4179152

[B111] Strauss N. Hendee E. D. (1959). The effect of diphtheria toxin on the metabolism of HeLa cells. J. Exp. Med. 109, 145–163. 10.1084/jem.109.2.145 13620845 PMC2136940

[B112] Stroke I. L. Letourneau J. J. Miller T. E. Xu Y. Pechik I. Savoly D. R. (2018). Treatment of *Clostridium difficile* infection with a small-molecule inhibitor of toxin UDP-glucose hydrolysis activity. Antimicrob. Agents Chemother. 62, e00107. 10.1128/AAC.00107-18 29483125 PMC5923158

[B113] Suh J. J. Pettinati H. M. Kampman K. M. O’Brien C. P. (2006). The status of disulfiram: a half of a century later. J. Clin. Psychopharmacol. 26, 290–302. 10.1097/01.jcp.0000222512.25649.08 16702894

[B114] Takehara M. Takagishi T. Seike S. Oda M. Sakaguchi Y. Hisatsune J. (2017). Cellular entry of *Clostridium perfringens* iota-toxin and Clostridium botulinum C2 toxin. Toxins 9, 247. 10.3390/toxins9080247 28800062 PMC5577581

[B115] Tam J. Hamza T. Ma B. Chen K. Beilhartz G. L. Ravel J. (2018). Host-targeted niclosamide inhibits *C. difficile* virulence and prevents disease in mice without disrupting the gut microbiota. Nat. Commun. 9, 5233. 10.1038/s41467-018-07705-w 30531960 PMC6286312

[B116] Tsuneoka M. Nakayama K. Hatsuzawa K. Komada M. Kitamura N. Mekada E. (1993). Evidence for involvement of furin in cleavage and activation of diphtheria toxin. J. Biol. Chem. 268, 26461–26465. 10.1016/s0021-9258(19)74337-3 8253774

[B117] Uddin T. M. Chakraborty A. J. Khusro A. Zidan B. R. M. Mitra S. Emran T. B. (2021). Antibiotic resistance in microbes: history, mechanisms, therapeutic strategies and future prospects. J. Infect. Public Health 14, 1750–1766. 10.1016/j.jiph.2021.10.020 34756812

[B118] Uematsu Y. Kogo Y. Ohishi I. (2007). Disassembly of actin filaments by botulinum C _2_ toxin and actin‐filament‐disrupting agents induces assembly of microtubules in human leukaemia cell lines. Biol. Cell. 99, 141–150. 10.1042/BC20060089 17067287

[B119] US Centers for Disease Control and Prevention (2019). Antibiotic resistance threats in the United States, 2019. Accessed August 08, 2024. 10.15620/cdc:82532

[B120] Vallari R. C. Pietruszko R. (1982). Human aldehyde dehydrogenase: mechanism of inhibition of disulfiram. Science 216, 637–639. 10.1126/science.7071604 7071604

[B121] Vandekerckhove J. Schering B. Bärmann M. Aktories K. (1988). Botulinum C2 toxin ADP-ribosylates cytoplasmic beta/gamma-actin in arginine 177. J. Biol. Chem. 263, 696–700. 10.1016/s0021-9258(19)35408-0 3335520

[B122] Van Ness B. G. Howard J. B. Bodley J. W. (1980a). ADP-ribosylation of elongation factor 2 by diphtheria toxin. Isolation and properties of the novel ribosyl-amino acid and its hydrolysis products. J. Biol. Chem. 255, 10717–10720. 10.1016/s0021-9258(19)70366-4 7000782

[B123] Van Ness B. G. Howard J. B. Bodley J. W. (1980b). ADP-ribosylation of elongation factor 2 by diphtheria toxin. NMR spectra and proposed structures of ribosyl-diphthamide and its hydrolysis products. J. Biol. Chem. 255, 10710–10716. 10.1016/s0021-9258(19)70365-2 7430147

[B124] Vitale G. Bernardi L. Napolitani G. Mock M. Montecucco C. (2000). Susceptibility of mitogen-activated protein kinase kinase family members to proteolysis by anthrax lethal factor. Biochem. J. 352 (Pt 3), 739–745. 10.1042/bj3520739 11104681 PMC1221512

[B125] Wesche J. Elliott J. L. Falnes P. Ø. Olsnes S. Collier R. J. (1998). Characterization of membrane translocation by anthrax protective antigen. Biochemistry 37, 15737–15746. 10.1021/bi981436i 9843379

[B126] Wickramage I. Spigaglia P. Sun X. (2021). Mechanisms of antibiotic resistance of *Clostridioides difficile* . J. Antimicrob. Chemother. 76, 3077–3090. 10.1093/jac/dkab231 34297842 PMC8598299

[B127] Wright C. Moore R. D. (1990). Disulfiram treatment of alcoholism. Am. J. Med. 88, 647–655. 10.1016/0002-9343(90)90534-K 2189310

[B128] Young J. A. T. Collier R. J. (2007). Anthrax toxin: receptor binding, internalization, pore formation, and translocation. Annu. Rev. Biochem. 76, 243–265. 10.1146/annurev.biochem.75.103004.142728 17335404

[B129] Zhu Z. Schnell L. Müller B. Müller M. Papatheodorou P. Barth H. (2019). The antibiotic bacitracin protects human intestinal epithelial cells and stem cell-derived intestinal organoids from *Clostridium difficile* toxin TcdB. Stem Cells Int. 2019, 4149762–4149768. 10.1155/2019/4149762 31467562 PMC6701344

