# Supplementary material for: Repurposing FDA-approved disulfiram for targeted inhibition of diphtheria toxin and the binary protein toxins of Clostridium botulinum and Bacillus anthracis
Source: Front Pharmacol. 2024 Sep 13;15:1455696. doi: 10.3389/fphar.2024.1455696 (PMC11427369; doi:10.3389/fphar.2024.1455696)
Supplement: Supplementary file 1 [file DataSheet1.PDF]

Supplementary Material

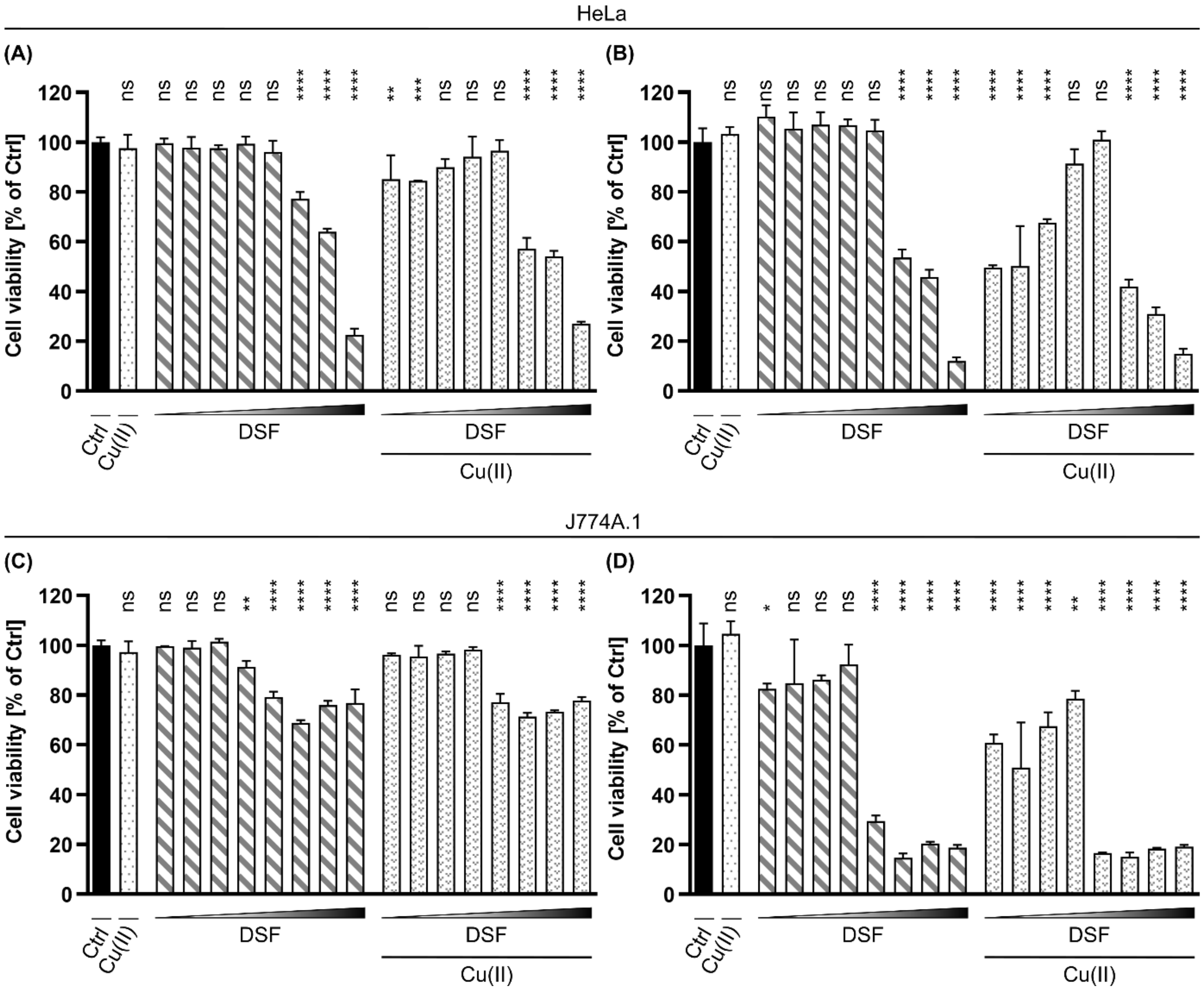

Supplementary Figure 1. Effect of DSF with or without Cu(II) on HeLa and J774A.1 cell viability. The relative viability (% of Ctrl) of cells treated with increasing concentrations of DSF (0.94 µM, 1.88 µM, 3.75 µM, 7.50 µM, 15 µM, 30 µM, 60 µM or 120 µM) in the presence or absence of 330 nM Cu(II) after 5 h **(A)** and **(C)** or 24 h **(B)** and **(D)** was measured via MTS assay. Values are given as mean ± SD (n=3) of triplicates from one representative experiment of three independent replicates. Statistical analysis was performed and all conditions were compared to the Ctrl condition by using one-way ANOVA with Dunnett's correction for multiple comparison (ns $p \geq 0.05$, * $p < 0.05$, ** $p < 0.01$, *** $p < 0.001$, **** $p < 0.0001$).

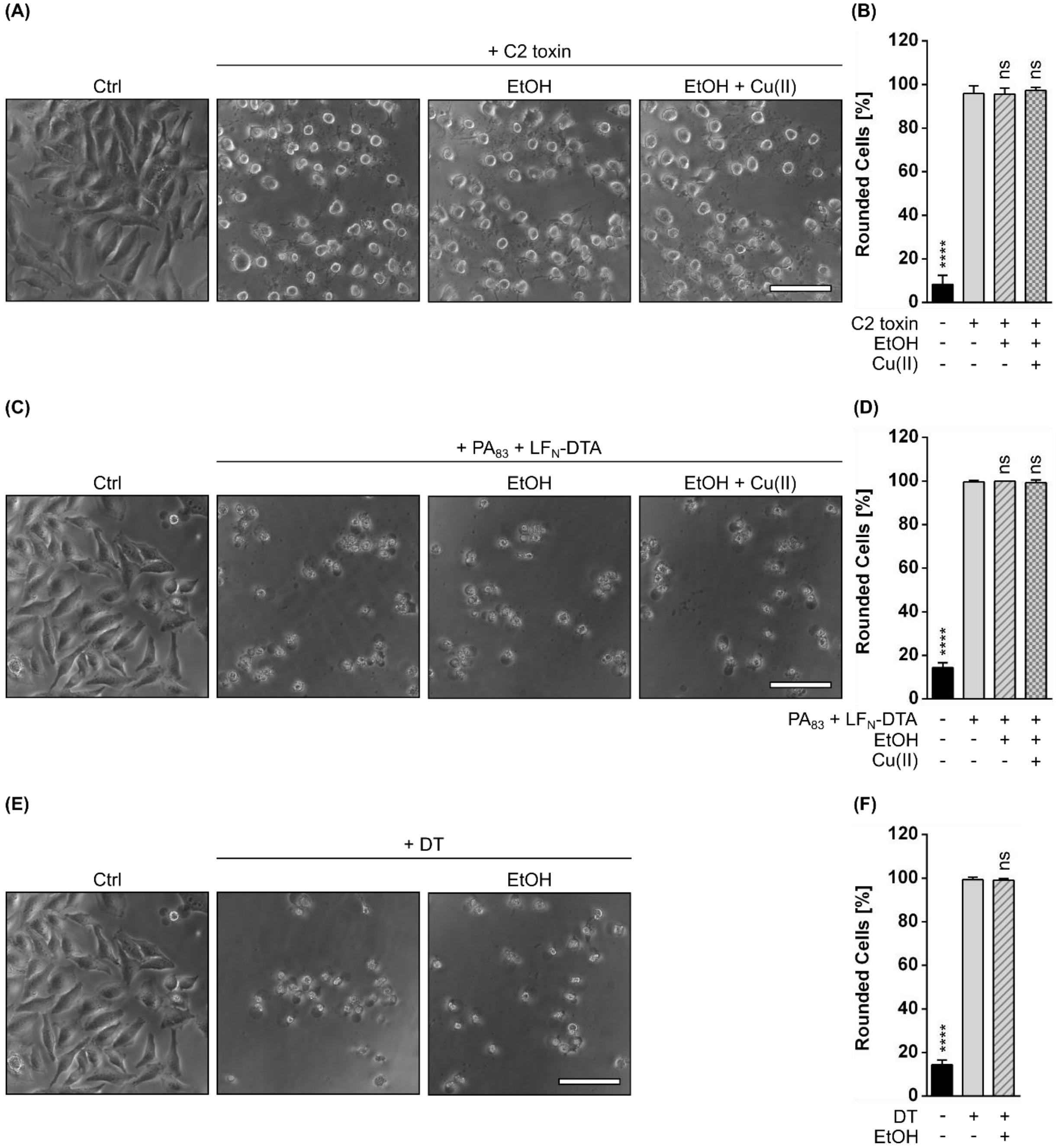

Supplementary Figure 2. The DSF solvent EtOH (in combination with or without Cu(II)) does not affect the intoxication of HeLa cells by C2 toxin, PA_{83} + LF_N-DTA or DT when it is present in a volume identical to 15 µM DSF (0.15% (v/v)). **(A)** Representative pictures after 5 h incubation time under the indicated conditions. Compound concentrations were 60 ng/mL C2I with 120 ng/mL C2IIa, 0.15% (v/v) EtOH and 330 nM Cu(II). Sale bar corresponds to 100 µm. **(B)** Quantitative evaluation of cell rounding after 5 h incubation with C2 toxin (60 ng/mL C2I + 120 ng/mL C2IIa), 0.15% (v/v) EtOH and 330 nM Cu(II) in the indicated combinations, or without any compound as control (Ctrl). Values are given as mean ± SD (n=3) of triplicates from one representative experiment of three independent replicates. Statistical analysis was performed and conditions were compared to the C2 toxin only condition by using one-way ANOVA with Dunnett's correction for multiple comparison (ns $p \geq 0.05$,

**** $p < 0.0001$). **(C)** Representative pictures after 24 h incubation time under the indicated conditions. Compound concentrations were 0.3 nM PA_{83} with 0.28 nM LF_N-DTA, 0.15% (v/v) EtOH and 330 nM Cu(II). Sale bar corresponds to 100 µm. **(D)** Quantitative evaluation of cell rounding after 24 h incubation with 0.3 nM PA_{83} + 0.28 nM LF_N-DTA, 0.15% (v/v) EtOH and 330 nM Cu(II) in the indicated combinations, or without any compound as control (Ctrl). Values are given as mean ± SD (n=3) of triplicates from one representative experiment of three independent replicates. Statistical analysis was performed and conditions were compared to the PA_{83} + LF_N-DTA only condition by using one-way ANOVA with Dunnett's correction for multiple comparison (ns $p \geq 0.05$, **** $p < 0.0001$). **(E)** Representative pictures after 24 h incubation time under the indicated conditions. Compound concentrations were 150 pM DT and 0.15% (v/v) EtOH. Sale bar corresponds to 100 µm. **(F)** Quantitative evaluation of cell rounding after 24 h incubation with 150 pM DT and 0.15% (v/v) EtOH in the indicated combinations, or without any compound as control (Ctrl). Values are given as mean ± SD (n=3) of triplicates from one representative experiment of three independent replicates. Statistical analysis was performed and conditions were compared to the DT only condition by using one-way ANOVA with Dunnett's correction for multiple comparison (ns $p \geq 0.05$, **** $p < 0.0001$). The data of (C), (D), (E) and (F) were obtained within the same experiment.
